# Evaluating meteorological data from weather stations, and from satellites and global models for a multi-site epidemiological study

**DOI:** 10.1016/j.envres.2018.02.027

**Published:** 2018-08

**Authors:** Josh M. Colston, Tahmeed Ahmed, Cloupas Mahopo, Gagandeep Kang, Margaret Kosek, Francisco de Sousa Junior, Prakash Sunder Shrestha, Erling Svensen, Ali Turab, Benjamin Zaitchik, The MAL-ED Network

**Affiliations:** a Department of International Health, Johns Hopkins Bloomberg School of Public Health, Baltimore, MD, USA; b Nutrition & Clinical Services Division, International Centre for Diarrhoeal Disease Research, Bangladesh (ICDDR,B), Dhaka, Bangladesh; c Department of Nutrition, University of Venda, South Africa; d Christian Medical College, Vellore, India; e Universidade Federal do Ceará, Brazil; f Department of Child Health, Institute of Medicine of Tribhuvan University, Kathmandu, Nepal; g Haukeland University Hospital, Norway; h Research and Development, Maternal and Child Health (MCH) Program, Karachi, Pakistan; i Department of Earth and Planetary Sciences, Johns Hopkins Krieger School of Arts and Sciences, Baltimore, MD, USA

**Keywords:** CHIRPS, Climate Hazards Group Infrared Precipitation with Stations, DD, Decimal degree, DISC, Data and Information Services Center, EID, Enteric infectious diseases, EO, Earth observation, FPR, False positive rate, GDAS, Global Data Assimilation System, GES, Goddard Earth Sciences, GLDAS, Global Land Data Assimilation System, LSM, Land Surface Model, MAL-ED, the Malnutrition & Enteric Infections: Consequences for Child Health and Development project, MBE, Mean bias error, NASA, National Aeronautics and Space Administration, NOAA, National Oceanic and Atmospheric Administration, NSE, Nash-Sutcliffe efficiency coefficient, R, Pearson's correlation coefficient, RMSE, Root mean square error, TPR, True positive rate, Meteorological data, Environmental epidemiology, Climate, Earth Observation data, Rotavirus

## Abstract

**Background:**

Longitudinal and time series analyses are needed to characterize the associations between hydrometeorological parameters and health outcomes. Earth Observation (EO) climate data products derived from satellites and global model-based reanalysis have the potential to be used as surrogates in situations and locations where weather-station based observations are inadequate or incomplete. However, these products often lack direct evaluation at specific sites of epidemiological interest.

**Methods:**

Standard evaluation metrics of correlation, agreement, bias and error were applied to a set of ten hydrometeorological variables extracted from two quasi-global, commonly used climate data products – the Global Land Data Assimilation System (GLDAS) and Climate Hazards Group InfraRed Precipitation with Stations (CHIRPS) - to evaluate their performance relative to weather-station derived estimates at the specific geographic locations of the eight sites in a multi-site cohort study. These metrics were calculated for both daily estimates and 7-day averages and for a rotavirus-peak-season subset. Then the variables from the two sources were each used as predictors in longitudinal regression models to test their association with rotavirus infection in the cohort after adjusting for covariates.

**Results:**

The availability and completeness of station-based validation data varied depending on the variable and study site. The performance of the two gridded climate models varied considerably within the same location and for the same variable across locations, according to different evaluation criteria and for the peak-season compared to the full dataset in ways that showed no obvious pattern. They also differed in the statistical significance of their association with the rotavirus outcome. For some variables, the station-based records showed a strong association while the EO-derived estimates showed none, while for others, the opposite was true.

**Conclusion:**

Researchers wishing to utilize publicly available climate data – whether EO-derived or station based - are advised to recognize their specific limitations both in the analysis and the interpretation of the results. Epidemiologists engaged in prospective research into environmentally driven diseases should install their own weather monitoring stations at their study sites whenever possible, in order to circumvent the constraints of choosing between distant or incomplete station data or unverified EO estimates.

## Highlights

• Earth observation data has potential for use in epidemiology but is seldom validated.
• The performance of two EO products varied depending on numerous factors.
• Epidemiologists should use their own weather stations to record meteorological data.
• Daily estimates of meteorological variables are associated with rotavirus infection.
• Choice of climate data source should be determined by the research question.

## 1 Introduction

Climate and weather influence population health through a number of interrelated pathways. Extreme weather events such as heatwaves, coastal floods and storm surges can both cause mortality directly and can compromise water sources and crop production, leading to widespread food and water insecurity, illness, undernutrition and other morbidities (World Health Organization, 2014). Moreover, climate is one of the primary constraints on the geographic and seasonal distribution of pollutants (Fann et al., 2016) and infectious agents (Wu et al., 2016). The growth, survival and dispersal of microorganisms and the viable range of their intermediary hosts and vectors is determined by environmental and hydrometeorological conditions (Hellberg and Chu, 2015). An increased awareness of the knowledge gaps surrounding these relationships, as well as the urgency of the climate change threat and greater understanding of its likely impact on public health has spurred calls for a research agenda to elucidate the interactions and biological mechanisms through which weather influences health (Xu et al., 2012, Rodó et al., 2013). A major barrier to this is the scarcity of empirical data linking climate and health at a sufficient level of spatiotemporal disaggregation for use in longitudinal and time series regression analyses (Kolstad and Johansson, 2011). To isolate interactions between the numerous, collinear climatic variables, quantify annual cycles and long-term trends, and incorporate lag effects, the health outcome and environmental exposure must be matched by their precise timing (Kolstad and Johansson, 2011, Hervás et al., 2014, Patel et al., 2013, Ahmed et al., 2013). Until recently, such analyses were hindered by the difficulty of accessing accurate and complete data on hydrometeorological predictors at high temporal resolution. The increased accessibility of Earth Observation (EO) climate data products – those derived from satellites and model-based reanalysis - is beginning to change this, but uptake has been slow due to a lack of interdisciplinary collaboration between the planetary sciences and public health fields (Rodó et al., 2013, Grace et al., 2015, Moore et al., 2017, Grace, 2017).

Researchers wishing to include climate variables as predictors in analyses of health outcomes generally have two options: to use either EO-derived or station-based data. The former have the advantage of completeness, both temporal and spatial. Estimates may be available at a daily or even sub-hourly resolution (Fang et al., 2009) without gaps and can be extracted for any location for which the geographical coordinates are known or a relevant geographic area can be mapped. Many also offer a larger suite of mutually consistent variables than are typically available from weather stations, and the data are often freely available to access online. Disadvantages include the wide variation in the uncertainty of the estimates (Hamm et al., 2015).

Weather conditions recorded at ground-based stations may be considered the gold standard for meteorological data, insofar as one exists, but are also subject to limitations. Lack of capacity to maintain routine record keeping may lead to significant data gaps, forcing researchers either to exclude outcome data for which no coincident exposure measures are available thus reducing statistical power, or to rely on summary measures such as moving mean values or binned aggregates, reducing variability and temporal resolution. Furthermore, weather stations are often situated in locations key to their primary uses in aviation or in monitoring weather for large population centers (i.e. cities and airports) and may be more geographically representative of some areas than others. Epidemiological surveillance sites may lie many kilometers from their nearest weather stations, distances greater than those over which localized meteorological conditions vary, introducing further error. Accessing data may be a challenge and, while the US National Oceanic and Atmospheric Administration (NOAA) offers a substantial online repository of historical data for some 9000 stations around the globe, for less well-served locations coordination with local meteorological agencies and organizations on the ground may be required (National Oceanic and Atmospheric Administration, 2016). Finally, weather stations vary in their accuracy and generally only record a small subset of variables – often only temperature, rainfall, pressure and wind speed - and more technically demanding measures, such as humidity and solar radiation, may be lacking.

The aim of this paper is to report on an exercise in selecting climate data products and assessing their performance both in characterizing meteorological conditions at the specific locations of epidemiological study sites and as predictors of a known climate-sensitive outcome – namely rotavirus infection episodes. The hypothesis that we aim to test is that gridded, EO-derived climate data products can be used as valid surrogates in longitudinal analyses where ground-based measurements are unavailable or incomplete to predict health outcomes at particular locations. As an illustrative case study, we use the eight study sites of the Malnutrition & Enteric Infections: Consequences for Child Health and Development (MAL-ED) project and focus on variables that we hypothesize to be associated with enteric infectious disease (EID) transmission (MAL-ED Network Investigators, 2014).

## 2 Material and methods

### 2.1 Site descriptions

**Fig. 1:**
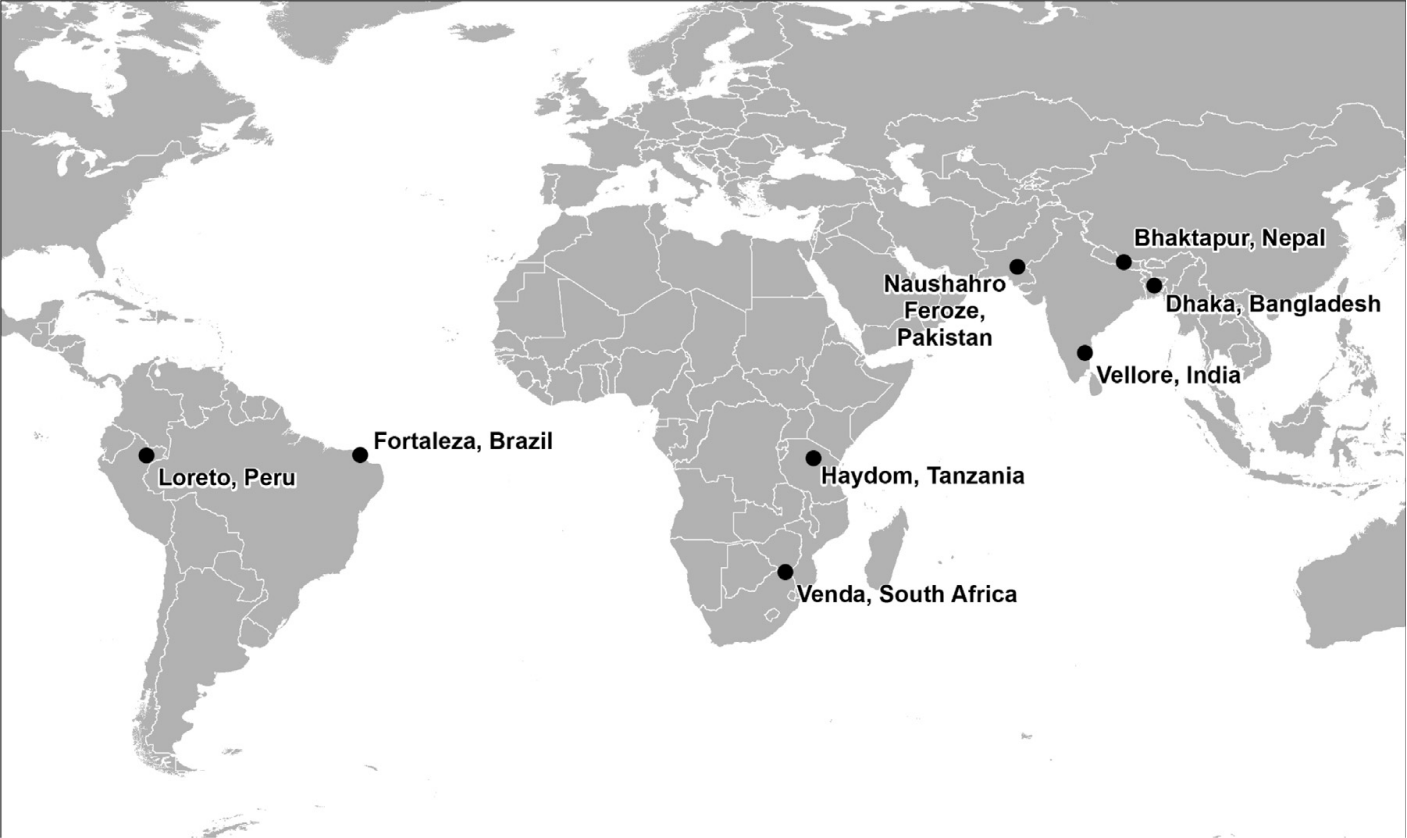
Locations of the eight MAL-ED sites.

The MAL-ED project was established in 2009 to investigate risk factors for enteric infection, diarrheal disease, undernutrition and other related adverse outcomes. This network of institutions recruited and monitored birth cohorts in eight communities, each in a different low- and middle-income country – Bangladesh, Brazil, India, Nepal, Pakistan, Peru, South Africa and Tanzania – across three continents. Table 1 summarizes the Köppen-Geiger climate classifications, precipitation and temperature patterns and other features of each of the MAL-ED study sites and Fig. 1 shows their locations. While the sites were originally selected to be characteristic of a variety of epidemiological contexts, they also vary in the type of climate that they experience, offering a representative range of the kinds of weather patterns that prevail across the developing regions of the world. Because they are situated at different latitudes and are divided equally between the northern and southern hemispheres, they also experience their rainy seasons and annual peaks in temperature at different times of the year and at different intensities. Similarly, the type of settlement and the altitude and topography of their locations – factors which may either have a direct effect on the weather they experience, or mediate the effect on EID incidence – all vary between sites. The MAL-ED project sites were selected as an illustrative example not only because they allow for the assessment of weather data quality and availability over a representative range of contexts, but also so that this information could be linked temporally and geographically with data on an outcome of public health importance.

**Table 1:** Köppen-Geiger climate classifications, precipitation and temperature patterns and other features of the locations of each MAL-ED study site (Institute for Vetinary Public Health, 2011, MAL-ED, 2015, Ahmed et al., 2014, Bessong et al., 2014, John et al., 2014, Lima et al., 2014, Mduma et al., 2014, Shrestha et al., 2014, Turab et al., 2014, Yori et al., 2014).

**Site**	**Main Climate**	**Precipitation**	**Temperature**	**Topography**	**Geographic extent of site (km)**		**Altitude (m)**	**Distance to weather station (km)**	**Settlement type**	**Hemisphere**
					**North-south**	**East-west**				
**Dhaka, Bangladesh**	Tropical savanna	Dry Nov - Feb,	Mar – May peak	Alluvial plain	0.8	0.6	12	4.6	Urban	Northern
		Monsoon Jun - Oct								
**Fortaleza, Brazil**	Tropical monsoon	Dry Aug - Dec,	Hot year-round	Coastal	1.5	1.2	28	5.3	Urban	Southern
		rainy Jan - July								
**Vellore, India**	Tropical savanna	Dry Jan - May,	Mar – Jun peak	Hilly	1.5	1.1	231	1.0	Urban	Northern
		Monsoon Jun - Dec								
**Bhaktapur, Nepal**	Humid subtropical	Dry Oct- Mar,	Hot during Monsoon,	Hilly	2.2	2.8	1317	7.5	Peri-urban	Northern
		Monsoon May - Aug	Apr – Jun							
**Naushero Feroze, Pakistan**	Desert/Arid	Dry, short Monsoon Jul -Sep	Very hot Mar – Oct	Flat	8.4	4.6	44	21.9	Rural	Northern
**Loreto, Peru**	Tropical rainforest	Fully humid,	Hot year-round	Flat	2.1	1.5	89	3.3	Rural	Southern
		year-round rain								
**Venda, South Africa**	Humid subtropical	Dry May - Sep, rainy Oct - Mar	Hot	Hilly	3.8	12.5	657	36.9	Peri-urban	Southern
			Sep – Feb							
**Haydom, Tanzania**	Tropical savanna	Dry Jun - Sep,	Temperate year round	Hilly	3.0	5.5	1650	31.8	Rural	Southern
		rainy Nov - May								

### 2.2 Hydrometeorological data

The first step in this analysis was to compile a list of hydrometeorogical variables that either have been demonstrated or are hypothesized to be associated with EID transmission in general and rotavirus incidence in particular. The following variables were selected: ambient temperature (D’Souza et al., 2008, Levy et al., 2009, Atchison et al., 2010, Barril et al., 2015, Jagai et al., 2012, Sumi et al., 2013), rainfall (Levy et al., 2009, Jagai et al., 2012, Sumi et al., 2013), humidity (D’Souza et al., 2008, Levy et al., 2009, Hashizume et al., 2008), atmospheric pressure (Hervás et al., 2014), wind (Hervás et al., 2014, Sumi et al., 2013), UV radiation (Hervás et al., 2014, D’Souza et al., 2008, Norval, 2006), soil moisture (Hurst et al., 1980) and water runoff (Barril et al., 2015). Next, available data sources were reviewed for daily estimates of these parameters for the eight MAL-ED study site locations over the period of follow-up.

#### 2.2.1 EO-derived data

Estimates of all the above variables are available from the Global Land Data Assimilation System (GLDAS – version 1). GLDAS derives meteorological fields from the Global Data Assimilation System (GDAS), an operational atmospheric analysis system that merges a global climate model – a numerical representation of the physical processes and energy fluxes occurring in the earth's atmosphere, oceans and land surfaces — with a diverse suite of in situ and satellite-derived observations (Intergovernmental Panel on Climate Change (IPCC) Working Group, 2013, Rodell et al., 2004). The system applies bias correction to GDAS precipitation and radiation estimates and employs the adjusted surface meteorology fields to drive advanced land surface models (LSMs) that simulate surface hydrological conditions. The GLDAS ensemble of LSMs includes the Noah LSM (Chen et al., 1996), which is implemented in GLDAS at a horizontal resolution of 0.25 decimal degrees (DDs) and parameterized with globally gridded maps of land surface parameters such as topography, land cover and soil texture classifications to produce near-real-time predictions available with global coverage and a temporal resolution of 3 h (Rodell et al., 2004, Goddard Earth Sciences Data and Information Services Center (GES DISC), 2016). Its products have been applied in numerous studies of climate, hydrology, agriculture, and ecology, as well as, more recently, public health outcomes (Grace et al., 2015, Moore et al., 2017). It is internally consistent across locations and between variables (although GLDAS version 1 can suffer from temporal discontinuities as input datasets change over time) (Kato et al., 2007). GLDAS data is disseminated as part of the mission of NASA's Earth Science Division and archived and distributed by the Goddard Earth Sciences (GES) Data and Information Services Center (DISC).

Although GLDAS does offer precipitation estimates, it employs a standard correction for bias in the GDAS precipitation field, whereas the Climate Hazards Group Infrared Precipitation with Stations (CHIRPS, version 2) product, which was developed solely to estimate rainfall, calibrates cloud-top temperature estimates and gauge-satellite data by interpolating observation data and weighting it according to proximity to the five closest weather stations (Funk et al., 2015). CHIRPS daily data has a resolution of 0.05 DDs (~5 km^2^) and has the potential to offer greater information content in poorly monitored areas and in tropical regions than alternative, entirely gauge-based products (Funk et al., 2015). Precipitation estimates from both sources were evaluated to determine the better-performing estimate. Neither GLDAS or CHIRPS products come with error estimates.

For both GLDAS and CHIRPS, a script was run to extract all variable values from the gridded files during the period 2009 – 2014 for the grid cells corresponding to the coordinates of the eight MAL-ED site locations. For the GLDAS variables, the 3-hourly estimates were aggregated to daily averages, totals or maximum and minimum as appropriate, while the daily estimated rainfall totals were taken from the CHIRPS product.

The following variables were extracted from the two gridded products.

• *Maximum and minimum daily temperatures in degrees Celsius* **–** Air temperature varies as a function of latitude, elevation, and large scale atmospheric circulations and is sensitive to prevailing weather patterns and differences in local surface conditions. Pathogens are only able to propagate within certain temperature ranges, so associations between ambient temperature and infectious disease risk could be related to the agents’ ability to survive in the environment (Naumova et al., 2007). A study comparing daily maximum and minimum land surface temperatures in GLDAS with data from GHCN weather stations across the globe found broad agreement both globally and separately for the regions in which the MAL-ED sites are located (Ji et al., 2015).
• *Daily total precipitation volume in millimeters* **–** Precipitation patterns vary on a very localized scale due to interactions between energy and water fluxes and features of land-sea geometry and topography (Wallace and Hobbs, 2006). Following heavy precipitation events, microorganisms that are able to survive in aquatic environments may be dispersed over large geographical areas in water runoff (Hellberg and Chu, 2015). Conversely, periods of drought and decreased rainfall have also been shown to be associated with increases in rates of diarrheal disease, thought to be due to water scarcity that leaves people reliant on unprotected water sources and unable to maintain hygiene-promoting behaviors (Lloyd et al., 2007). CHIRPS rainfall estimates have been shown to correlate well with in situ precipitation records across South America and West Africa for annual rainfall totals (Ceccherini et al., 2015), across East Africa for springtime averages (Funk et al., 2013, Shukla et al., 2014), in Mozambique for dekadal totals (Toté et al., 2015) and in Cyprus for monthly totals (Katsanos et al., 2016).
• *Surface pressure (Pa)* **-** High surface pressure is frequently associated with still, clear sky conditions, in which mixing of air masses is slow, while low pressure occurs under convective, stormy conditions when winds are high. Such conditions may favor rapid diffusion of airborne particles, including infectious agents, though they may also be associated with rainfall events that scavenge airborne particles from the air (Hervás et al., 2014).
• *Wind speed (m/s)* **-** Wind is a possible means of dispersal of virally infective dried fecal particles and for spore-forming bacteria (Hellberg and Chu, 2015). Using a meteorological standard, GLDAS reports estimates of 10 m height winds as an indicator of broader conditions.
• *Humidity* **–** Humidity is thought to be inversely associated with transmission risk for numerous viruses, which may relate to the conditions conducive to the survival of the shed virions outside the host, or to their areal transport in dried, infective fecal and dust particles (Levy et al., 2009). Two measures of humidity are widely used in climate science and so were included in this analysis:
  ○ *Specific humidity in kilograms of water per kilogram of air (kg/kg)* **-** Near-surface specific humidity – the moisture content of the air – is closely related to temperature, distribution of surface water, soil moisture, and evapotranspiration, and so seasonal and zonal variations vary closely alongside these parameters. GLDAS-derived estimates of specific humidity have been demonstrated to have near-perfect correlation (r = 0.98) with in situ meteorological observations for a particular location in Northeast China (Qi et al., 2015).
  ○ *Relative humidity (%)* **–** Expressed as a percentage, relative humidity is the ratio of the specific humidity to the saturation specific humidity and is the measure more commonly used in research on determinants of pathogen survival and transmission (Sattar et al., 1984, Ijaz et al., 1985).
• *Solar radiation (W/m^2^)* **–** The amount of solar radiation that reaches the Earth's surface is highly indicative of cloud cover, once the Earth's position in its elliptical orbit around the sun and the tilt in its rotation axis are taken into account (Marshall and Alan, 2007). Higher levels of solar radiation may reduce transmission of pathogens in the outdoor environment due to the effect of UV radiation inactivating some viruses (D’Souza et al., 2008, Nuanualsuwan et al., 2002), and impeding the survival of some bacteria (Wu et al., 2016). Published site-specific evaluation of GLDAS solar radiation estimates are limited, though one study did show good correlation between GLDAS estimates at 3-h intervals with recordings from a ground-based flux measurement instrument in South Korea (r = 0.81) (Kim et al., 2016).
• *Soil moisture (%)* **–** The moisture content of soil is hypothesized to influence the survival of enteric pathogens in the environment (Hurst et al., 1980, Lal et al., 2012). Evaluation of GLDAS soil moisture estimates is also limited, particularly in subtropical and tropical areas. A study of GLDAS estimates of soil moisture using the Noah LSM showed good correlation with station-averaged surface soil moisture data for a for 20–40 cm layer on the central Tibetan Plateau, and another showed excellent correlation with data from a station in South Korea (r = 0.94) (Kim et al., 2016, Chen et al., 2013).
• *Surface runoff in millimeters* **–** The rate at which water drains following precipitation events may affect how microorganisms are dispersed over the landscape (Hellberg and Chu, 2015). Increased sewage outflows and runoff volumes – particularly following droughts - increase water turbidity causing pathogens from the sediment to re-suspend in surface water bodies, processes that may explain seasonal upticks in waterborne diseases (Hellberg and Chu, 2015, Lal et al., 2012). Modelled estimates of surface runoff are challenging to validate since field measurements of this parameter are sparse. However, one study used GLDAS runoff estimates and a source-to-sink river routing scheme to model river discharge at river gauge locations for major basins across the globe with performance varying by region and by LSM (Zaitchik et al., 2010).

#### 2.2.2 Weather station data

In the next stage, sources of ground-based observational data were sought that contained equivalent variables to the EO-derived measured at the nearest weather station to each MAL-ED site and covering as much of the MAL-ED follow-up period (2009 – 2014) as was available. To maintain consistency between sites, only the one nearest weather station to each site was considered. The data were either retrieved from NOAA's NNDC Climate Data Online repository (National Oceanic and Atmospheric Administration, 2016), if there was a National Climatic Data Center-contributing station close to the site, or otherwise were acquired from local meteorological authorities in coordination with site staff. Six of the sites had data available from NOAA for a nearby weather station, and for four of these – Bangladesh, Brazil, Nepal and Peru – the station was located within 7 km of the study site (Table 1). The nearest weather station to the study site in Pakistan was situated 22 km away, while the equivalent distance for the site in South Africa was 37 km, a scale that is likely to introduce error which should be taken into account when interpreting the results. The following variables were available from the NOAA database (National Climatic Data Center - Climate Services Branch, 2006):

• Maximum and minimum temperature for the day (degrees Fahrenheit to tenths)
• Total precipitation (rain and/or melted snow) reported during the day (inches to hundredths)
• Mean station pressure for the day (millibars to tenths)
• Mean wind speed for the day (knots to tenths)

While NOAA data was not available for the India site during the period of interest, similar data were obtained from the India Meteorological Department from a weather station located approximately 1 km from the study site, which included maximum and minimum daily temperature (°C), rainfall (mm) and relative humidity at 8:30 a.m. and at 17:30 p.m. Indian Standard Time, but did not include pressure or wind speed. In addition to the variables in the NOAA data, estimates of relative humidity (%) were obtained from the Pakistan Meteorological Department from a station at the same location as the NOAA-contributing station at 0:00 a.m. and 12:00 p.m. UTC (5:00 a.m. and 5:00 p.m. Pakistan Standard Time). Similarly, site staff in South Africa were able to provide hourly estimates of relative humidity from local authorities for the same station used by NOAA. In Tanzania there were no NOAA weather stations within 260 km of the study sites, and the only daily weather data that site staff were able to obtain for nearby were hand-written daily rainfall records from a farm located 32 km from the site, which, because they were used in routine monitoring of crop pests, only covered the armyworm moth season running from November to May coinciding with the rainy season. Qualitative reports from site staff indicated that conditions at this farm are slightly drier than at the study site itself. Despite its limitations, this information was digitized and included in the validation exercise.

Temperature and humidity at the South Africa weather station were measured using a Vaisala HUMICAP probe HMP45 D, which has an accuracy at 20 °C of ± 0.2 °C and ± 2%^1^ (Vaisala, 2006) while precipitation was measured using a tipping bucket rain gauge. No information was available regarding the equipment used at the other weather stations or the limits of uncertainty, distance at which they are believed to be accurate or expected variograms of the climate parameters. All NOAA-contributing weather stations are required to use equipment that conforms to the World Meteorological Association's general meteorological standards and recommended practices (World Meteorological Organization, 2015).

### 2.3 Evaluation criteria

Numerous statistical metrics are commonly used to assess the performance of EO-derived estimates of hydrometeorological parameters relative to ground- or station-based measurements. The metrics chosen for this study were:

• Pearson's correlation coefficient (*R*) – This measure of linear dependence between two variables is widely used and easily interpreted, taking a value between − 1 and 1 with 1 indicating perfect positive linear correlation (Kirkwood and Sterne, 2001).
• Nash-Sutcliffe efficiency coefficient (NSE) – This normalized indicator of model efficiency corresponds to the statistical agreement or skill of the estimates relative to the observed measurements and takes a value ranging from minus infinity to one, with one being a perfect fit and negative values meaning that the station mean offers a better estimate (Ji et al., 2015, Toté et al., 2015, Nash and Sutcliffe, 1970, Wang et al., 2011, Ahmed et al., 2016).
• Mean bias error (MBE) – This measures the extent to which the estimated value deviates from the observed value (de Oliveira et al., 2016). It can take any value, with negative values indicating systematic under-estimation and positive values, over-estimation, and zero indicating a perfect lack of bias (Wang et al., 2011, de Oliveira et al., 2016).
• Root mean square error (RMSE) – This is an absolute measure of the overall error in the estimates relative to the observed values, expressed in the same units and scale as the data itself (de Oliveira et al., 2016, Hyndman and Koehler, 2006). It can take any positive value with zero indicating a perfect lack of error (Wang et al., 2011).

In addition, to assess the ability of the EO data to characterize to extremes of each parameter, the following metrics were calculated with respect to days in which the parameter value measured by the weather stations exceeded the 80th percentile of the overall distribution of that parameter:

• True positive rate (TPR) – The proportion of days classified as extreme (> 80th percentile) for a particular parameter by the weather station that were also classified as such by the EO data (equivalent to the sensitivity of a diagnostic test in epidemiology).
• False positive rate (FPR) – The proportion of days not classified as extreme by the weather station that were nevertheless classified as such by the EO data (equivalent to 1 – sensitivity).

### 2.4 Rotavirus data

To assess the relative ability of the variables and datasets to predict an outcome that has been demonstrated to be climate dependent, they were merged with data on rotavirus infection from the MAL-ED cohorts. These data and the methods by which they were obtained have been described in detail elsewhere (Colston et al.,, Mohan et al., 2017). In brief, stool samples were collected from the study subjects on a monthly basis and upon reporting of a diarrheal episode from age 0 − 24 months and were tested for the presence of shed rotavirus by enzyme-linked immunosorbent assay (Houpt et al., 2014). The outcome is therefore a binary infection status variable indicating whether each child was positive or negative for rotavirus on each date on which a sample was collected (Colston et al., 2018).

### 2.5 Statistical analysis

All variables that were not in metric units were converted to their metric equivalents. The median daily temperatures were calculated from the maximum and minimum daily temperatures for both the station-based and gridded datasets. For Pakistan and India, the observed average daily relative humidity was approximated by taking the average of the station-based estimates for the two times that were available, whereas for South Africa, the daily averages of the hourly estimates were used. Surface pressure was expressed in millibars. The EO-derived data were found to be stable throughout the 2009–2014 period considered in this study at all sites and for all variables, with the exception of a slight discontinuity in the GLDAS surface pressure at the Tanzania site. This discontinuity in the original data was adjusted for in the data presented here by adding a simple offset to the second half of the record to align it with the previous period. A small number of implausible outlying values were dropped from the full dataset.^2^

As an initial exploration of the data, each of the hydrometeorological variables was plotted in time series alongside the station-based equivalents where available for each site. Next, the EO-derived values were each plotted against their station-based equivalents in scatterplots to visualize the fit between the two. Then, the evaluation metrics were calculated separately for each variable and site, first for the raw daily values and then for the mean of the values over seven days to determine whether averaging over this period improved the performance of the variables. As a basic method for evaluating the products in the absence of a seasonal cycle, these values were then recalculated after restricting the data to only the site-specific season of peak rotavirus transmission (for sites that experienced multiple peaks in transmission during the annual cycle, the primary peak of highest amplitude was used). This was to control for the sensitivity of the evaluation metrics to seasonal variation. We repeated the analysis for data extracted from the gridded products at the locations of the weather stations rather than those of the study sites and provide the results in supplementary tables for readers who are interested in location-specific spot checks. However, the main analysis compared EO-derived data extracted from the exact site location with stations within varying proximity in order to reflect the scenario realistically faced by epidemiologists in which a study site may be located some distance from its nearest weather station.

Finally, to test the relative ability of each data source and variable to predict a climate-sensitive health outcome, logistic regression models were fitted to rotavirus infections status across all sites combined using generalized estimating equations (GEE) with each of the meteorological variables in turn as the main exposure, lagged by 3 days (representing the estimated 2 day incubation period (Lee et al., 2013), plus 24 h to report symptoms). In all models, the main association was adjusted for age in continuous months, seasonality - using annual and biannual Fourier series functions to account for multiple peaks within the year (Colston et al., 2018) – and calendar time, each with site-specific interactions. For each hydrometeorological variable, the model was fitted first using the EO-derived data then, where available, the weather station data and third, a combination of the two in which missing station data was substituted with its EO-derived equivalent and compared results between daily and 7-day mean values. The purpose of this was to assess the sensitivity of the prediction models to differences in the data sources and period of aggregation. Odds ratios for these associations are reported alongside their 95% confidence intervals. Potential non-linearity, distributed lag effects, mediation and interaction among variables will be explored in subsequent MAL-ED publications but were beyond the scope of this paper. Analyses were carried out using Stata 13.1 (StataCorp, 2013). The merged data file is provided with the supplementary material (excluding the relative humidity measurements from the Pakistan and South Africa weather stations, which were provided on the understanding that they would not be shared with third parties).

## 3 Results

As shown in Table 1, three of the sites are located in tropical savannah climate zones (Bangladesh, India and Tanzania), while two share a humid subtropical climate (South Africa and Nepal). The maximum distance over which any site extends is 12.5 km (South Africa east to west) with most sites extending less than 5 km in any direction and, according to accounts from site staff, none of the sites exhibit large topographic contrasts. It was therefore assumed that within-site climate variation would be small, though localized environmental risk factors for specific households—e.g. small depressions or local water bodies—cannot be ruled out. Table 2 summarizes the nine hydrometeorological variables for the eight MAL-ED sites, while time-series plots of each variable by source are provided in the supplementary material (supplementary figs. 1–10). The weather station data for Bangladesh had the most missing data of the NOAA datasets, with just under 60% of the daily estimates for the period available for each of the four variables - temperature, precipitation, pressure and wind speed. The remaining NOAA datasets had fairly complete (> 90%) data on temperature and wind speed, while precipitation data were only below 90% completeness in Nepal and South Africa. Daily data on surface pressure were extremely sparse (~1%) for Nepal and Pakistan (estimates only available for 25 and 21 days respectively) and somewhat incomplete for Bangladesh and Brazil (59.1% and 70.0% respectively). The data on relative humidity from the local meteorological authorities were fully complete for Pakistan, and fairly complete for India, but only somewhat complete for South Africa. As previously described, the only station-based variable available for Tanzania was precipitation. These data were only available for 36.4% of the days in the period of interest, representing only the four November to May rainy seasons from 2010 to 2014. No in situ data on specific humidity, solar radiation, soil moisture or surface runoff were available for any of the 8 sites. These findings serve to underscore the fact that weather station data varies widely in scope, completeness, accessibility and spatial resolution.

**Table 2:** Summary of GLDAS, CHIRPS and weather-station-based hydrometeorological variables for the eight MAL-ED study sites, 2009–2014^a^.

**Variable**		**Median temperature (C)**		**Precipitation (mm)**			**Surface pressure (mbar)**		**Wind speed (m/s)**	
		**GLDAS**	**Station**	**GLDAS**	**CHIRPS**	**Station**	**GLDAS**	**Station**	**GLDAS**	**Station**
**BGD**	Median	27.9	28.8	0.0	0.0	0.0	1007.3	1005.7	2.6	0.6
	IQR	6.3	5.5	4.3	6.1	1.8	8.9	8.6	1.4	0.8
	Maximum	35.0	33.9	107.1	103.5	150.1	1019.6	1018.3	8.3	7.3
	Minimum	14.1	13.8	0.0	0.0	0.0	993.5	992.8	0.6	0.0
	Completeness	100.0%	59.7%	100.0%	100.0%	59.6%	100.0%	59.1%	100.0%	59.3%
**BRF**	Median	27.1	27.2	0.0	0.0	0.0	996.9	1008.7	5.0	4.9
	IQR	1.4	1.3	1.7	0.0	1.0	2.7	2.3	1.7	2.3
	Maximum	29.4	29.8	55.0	118.0	147.1	1002.2	1014.0	7.8	9.7
	Minimum	23.3	22.5	0.0	0.0	0.0	991.5	1003.8	0.9	1.1
	Completeness	100.0%	99.8%	100.0%	100.0%	99.0%	100.0%	70.0%	100.0%	99.8%
**INV**	Median	28.0	28.9	0.0	0.0	0.0	978.0	–	2.4	–
	IQR	5.8	5.1	2.9	4.0	0.2	5.6	–	1.0	–
	Maximum	37.0	36.5	70.7	72.5	88.4	986.8	–	6.2	–
	Minimum	19.6	20.9	0.0	0.0	0.0	968.4	–	0.8	–
	Completeness	100.0%	84.5%	100.0%	100.0%	84.8%	100.0%	–	100.0%	–
**NEB**	Median	17.8	20.5	0.0	0.0	0.0	837.6	862.1	1.8	1.4
	IQR	8.5	9.5	2.1	0.0	0.0	4.7	2.5	1.0	0.8
	Maximum	25.2	29.0	76.5	142.0	134.6	847.8	868.7	4.5	5.1
	Minimum	1.3	5.5	0.0	0.0	0.0	828.7	856.1	0.7	0.0
	Completeness	100.0%	97.6%	100.0%	100.0%	83.5%	100.0%	1.1%	100.0%	97.6%
**PKN**	Median	31.8	29.2	0.0	0.0	0.0	1003.1	994.8	2.5	0.9
	IQR	14.4	12.8	0.0	0.0	0.0	14.2	12.7	1.8	0.8
	Maximum	42.8	40.5	76.9	24.0	119.9	1019.0	1015.6	9.7	6.3
	Minimum	12.1	7.5	0.0	0.0	0.0	986.2	985.2	0.5	0.0
	Completeness	100.0%	99.0%	100.0%	100.0%	98.9%	100.0%	1.0%	100.0%	99.0%
**PEL**	Median	26.2	27.4	4.4	0.0	0.8	997.2	999.8	1.0	1.2
	IQR	1.9	1.4	11.5	11.2	7.4	3.1	3.0	0.3	0.7
	Maximum	30.7	30.6	88.9	84.1	199.9	1005.4	1009.4	2.1	3.5
	Minimum	20.4	18.1	0.0	0.0	0.0	991.1	993.1	0.3	0.0
	Completeness	100.0%	100.0%	100.0%	100.0%	90.9%	100.0%	94.0%	100.0%	94.6%
**SAV**	Median	19.3	21.9	0.0	0.0	0.0	910.6	946.5	2.1	2.4
	IQR	5.4	5.8	0.0	0.0	0.3	5.9	6.8	1.2	0.8
	Maximum	29.3	29.9	88.7	150.2	136.9	927.5	965.0	6.4	7.5
	Minimum	8.1	9.9	0.0	0.0	0.0	898.0	932.7	0.5	1.0
	Completeness	100.0%	96.1%	100.0%	100.0%	88.2%	100.0%	94.9%	100.0%	96.1%
**TZH**	Median	18.0	–	0.0	0.0	0.0	827.3	–	2.9	–
	IQR	2.0	–	0.4	0.0	0.0	2.0	–	1.3	–
	Maximum	22.6	–	46.5	47.7	38.5	831.4	–	5.8	–
	Minimum	13.4	–	0.0	0.0	0.0	823.4	–	0.6	–
	Completeness	100.0%	–	100.0%	100.0%	36.4%	100.0%	–	100.0%	–

**Variable**		Relative humidity (%)		Specific humidity (kg/kg)		Solar radiation (W/m^2^)		Soil moisture (%)		Surface runoff (mm)	
		**GLDAS**	**Station**	**GLDAS**	**Station**	**GLDAS**	**Station**	**GLDAS**	**Station**	**GLDAS**	**Station**
**BGD**	Median	71.7	–	0.016	–	202.8	–	30.2	–	0.0	–
	IQR	23.2	–	0.010	–	64.9	–	8.9	–	0.0	–
	Maximum	97.2	–	0.024	–	298.4	–	39.8	–	36.1	–
	Minimum	23.1	–	0.003	–	15.3	–	19.1	–	0.0	–
	Completeness	100.0%	–	100.0%	–	100.0%	–	100.0%	–	100.0%	–
**BRF**	Median	72.7	–	0.016	–	259.6	–	17.0	–	0.0	–
	IQR	8.0	–	0.002	–	61.9	–	8.5	–	0.0	–
	Maximum	92.1	–	0.019	–	338.9	–	38.4	–	7.7	–
	Minimum	58.6	–	0.012	–	45.8	–	13.2	–	0.0	–
	Completeness	100.0%	–	100.0%	–	100.0%	–	100.0%	–	100.0%	–
**INV**	Median	64.3	69.0	0.014	–	238.7	–	23.8	–	0.0	–
	IQR	21.8	19.5	0.003	–	70.4	–	13.1	–	0.0	–
	Maximum	95.8	99.0	0.020	–	311.1	–	41.6	–	31.2	–
	Minimum	29.1	36.5	0.006	–	15.0	–	12.1	–	0.0	–
	Completeness	100.0%	84.6%	100.0%	–	100.0%	–	100.0%	–	100.0%	–
**NEB**	Median	62.6	–	0.007	–	222.9	–	18.7	–	0.0	–
	IQR	42.5	–	0.009	–	96.1	–	12.9	–	0.0	–
	Maximum	97.5	–	0.017	–	350.7	–	37.1	–	17.4	–
	Minimum	4.6	–	0.001	–	12.8	–	9.9	–	0.0	–
	Completeness	100.0%	–	100.0%	–	100.0%	–	100.0%	–	100.0%	–
**PKN**	Median	27.3	63.5	0.006	–	235.5	–	7.8	–	0.0	–
	IQR	16.6	13.5	0.009	–	98.5	–	3.3	–	0.0	–
	Maximum	84.5	98.0	0.021	–	322.5	–	33.6	–	15.8	–
	Minimum	4.0	27.0	0.001	–	6.9	–	6.6	–	0.0	–
	Completeness	100.0%	100.0%	100.0%	–	100.0%	–	100.0%	–	100.0%	–
**PEL**	Median	87.3	–	0.018	–	191.6	–	34.1	–	0.0	–
	IQR	9.8	–	0.002	–	86.3	–	2.1	–	0.2	–
	Maximum	98.1	–	0.021	–	313.4	–	38.2	–	27.5	–
	Minimum	54.5	–	0.011	–	14.8	–	28.0	–	0.0	–
	Completeness	100.0%	–	100.0%	–	100.0%	–	100.0%	–	100.0%	–
**SAV**	Median	75.2	67.0	0.010	–	222.0	–	14.6	–	0.0	–
	IQR	21.0	19.0	0.005	–	98.7	–	4.8	–	0.0	–
	Maximum	98.0	97.0	0.018	–	362.2	–	31.6	–	3.4	–
	Minimum	16.2	15.0	0.002	–	9.2	–	9.2	–	0.0	–
	Completeness	100.0%	74.4%	100.0%	–	100.0%	–	100.0%	–	100.0%	–
**TZH**	Median	77.0	–	0.011	–	275.8	–	15.5	–	0.0	–
	IQR	17.1	–	0.003	–	57.6	–	6.7	–	0.0	–
	Maximum	97.7	–	0.015	–	348.2	–	29.8	–	5.9	–
	Minimum	38.6	–	0.006	–	35.1	–	11.7	–	0.0	–
	Completeness	100.0%	–	100.0%	–	100.0%	–	100.0%	–	100.0%	–

a BGD = Dhaka, Bangladesh; BRF = Fortaleza, Brazil; INV = Vellore, India; NEB = Bhaktapur, Nepal; PKN = Naushero Feroze Pakistan; PEL = Loreto, Peru; SAV = Venda, South Africa; TZH = Haydom, Tanzania; IQR = Inter-quartile range.

**Fig. 2:**
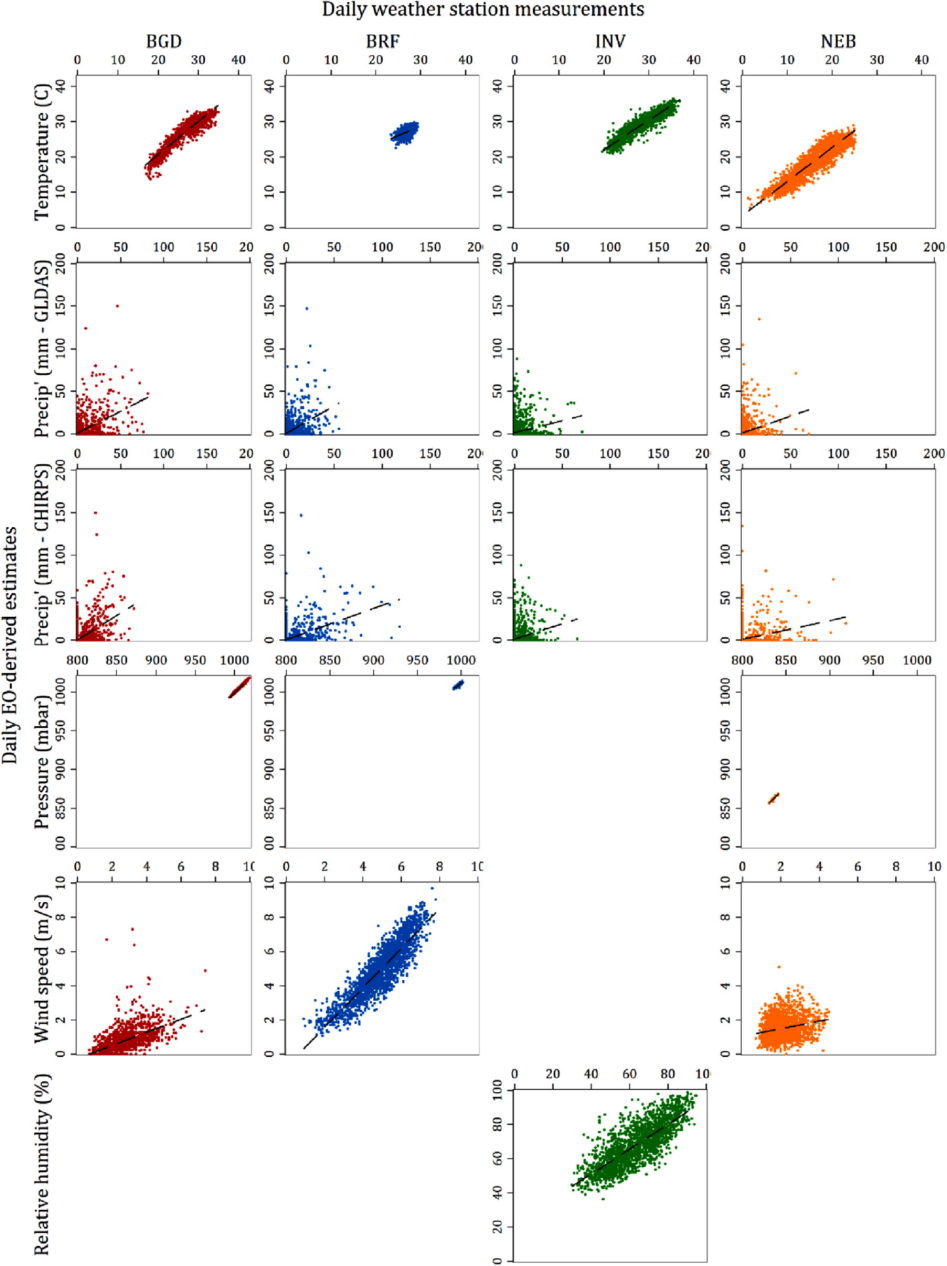

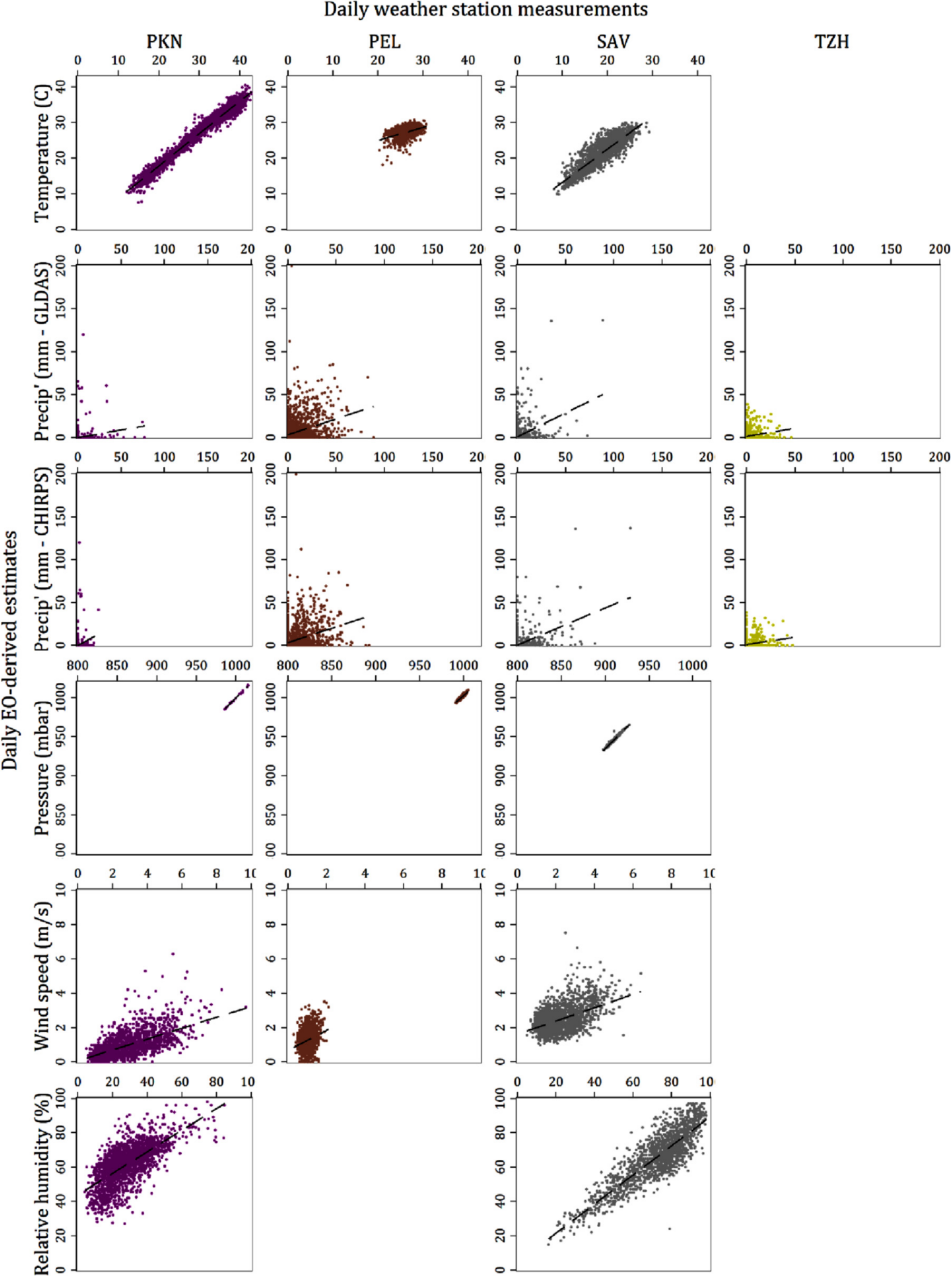
a: Scatter plot matrix of EO-derived daily variable estimates against station-based equivalents. b: Scatter plot matrix of EO-derived daily variable estimates against station-based equivalents.

**Fig. 3:**
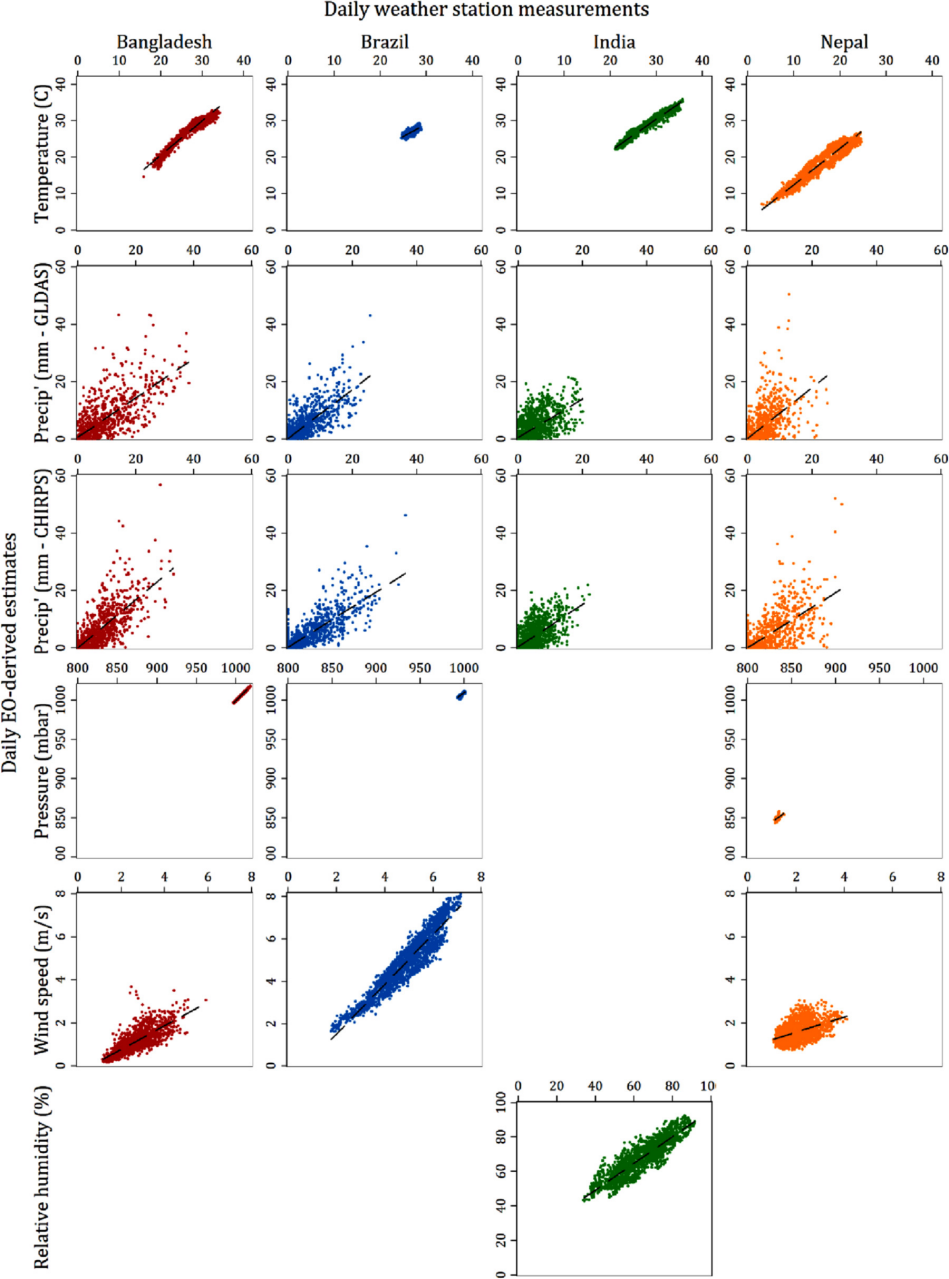

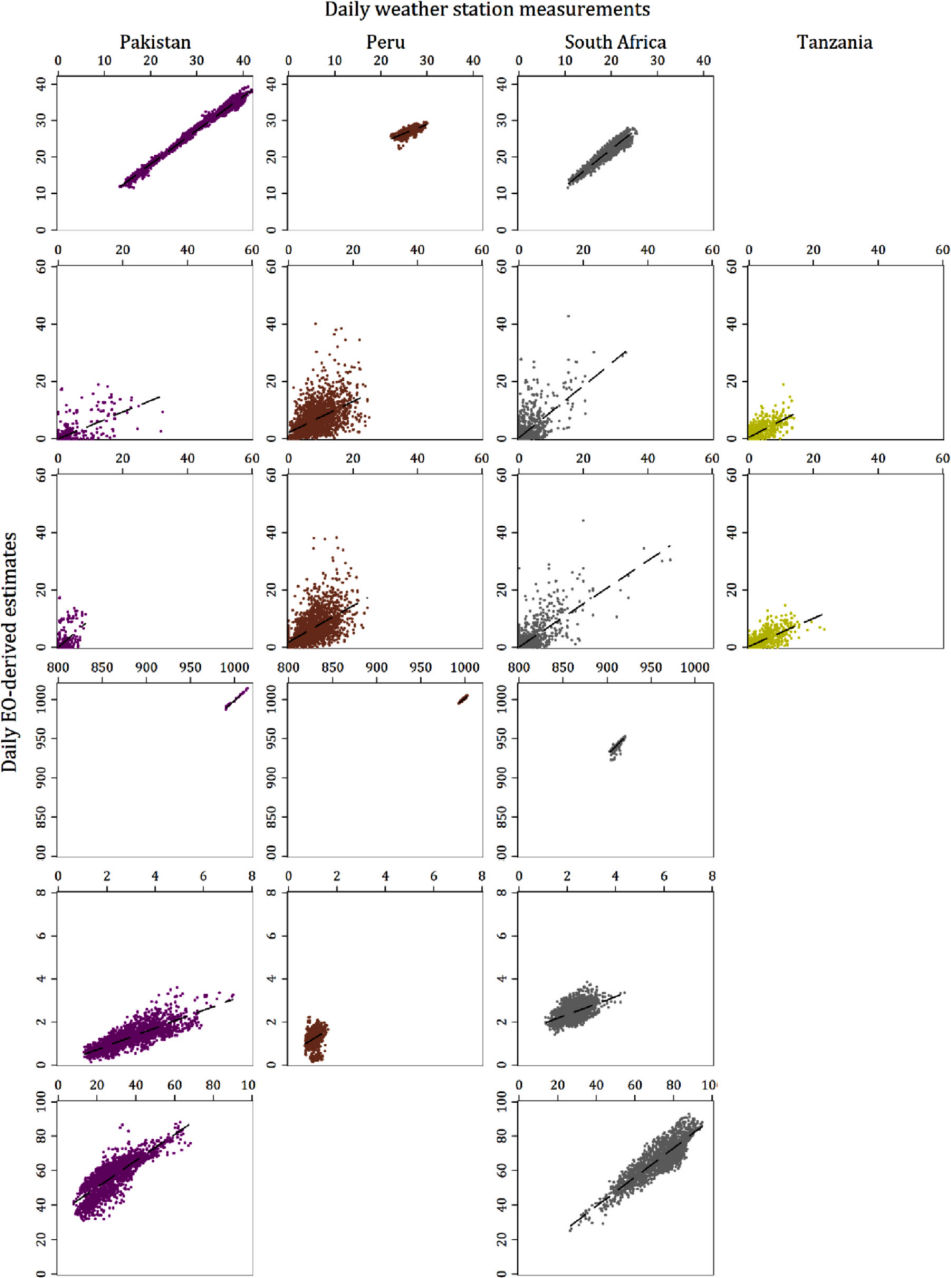
a: Scatter plot matrix of EO-derived 7-day average variable estimates against station-based equivalents. b: Scatter plot matrix of EO-derived 7-day average variable estimates against station-based equivalents.

Table 3 summarizes the evaluation metrics for both the daily estimates and the 7-day averages of all variables and sites for which both weather station and EO-derived data were available, and Fig. 2a and b shows scatter plots of the daily variable values from the two sources plotted against each other, while Fig. 3a and b shows the same for the values aggregated to 7-day means. Table 4 reports the results of the same evaluation metric calculations when the analysis was restricted only to the months of the year during which rotavirus has been found to be highest (Colston et al., 2018). Table 5 presents the odds ratios for the associations with rotavirus of each hydrometeorological variable calculated from logistic models fitted with GEE to the pooled (all-site) MAL-ED data over a 3-day lag adjusting for covariates. The columns indicate the source (weather station, EO or combined) and period of aggregation (daily or 7-day mean) of the meteorological predictor. The tables provided in the supplementary material present the equivalent statistics when the locations of the weather stations, rather than the study sites were used to extract data from the gridded products. For several sites, the GLDAS results are identical since both the site and its corresponding station were close enough that they fell within the same 0.25 DD grid square. In sites where these distances were larger, the difference in the results were mostly minor, with the notable exception of surface pressure in South Africa, for which the NSE, MBE and RMSE improved substantially.

**Table 3:** Evaluation statistics for hydrometeorological variables in the eight MAL-ED sites^a^.

**Variable**		**BGD**		**BRF**			**INV**		**NEB**				**PKN**			**PEL**			**SAV**		**TZH**		
		**Daily**	**7-day average**	**Daily**		**7-day average**	**Daily**	**7-day average**	**Daily**		**7-day average**		**Daily**		**7-day average**	**Daily**		**7-day average**	**Daily**	**7-day average**	**Daily**		**7-day average**
**Median temperature (C)**	**n**	1307			2186		1851		2138			2170				2190			2106		–		
	**R**	0.93	0.95		0.58	0.68	0.93	0.97	0.93	0.95		0.98		0.99		0.45	0.59		0.88	0.95	–	–	
	**NSE**	0.85	0.89		0.05	0.24	0.76	0.84	0.55	0.58		0.79		0.80		−1.22	−2.28		0.29	0.24	–	–	
	**MBE**	0.27	0.28		0.13	0.13	0.86	0.86	2.89	2.89		−3.02		−3.02		1.11	1.11		2.63	2.63	–	–	
	**RMSE**	1.54	1.27		0.88	0.65	1.58	1.25	3.51	3.32		3.37		3.22		1.82	1.43		3.23	2.83	–	–	
	**TPR**	0.28	0.27		0.51	0.59	0.51	0.54	0.52	0.55		0.78		0.83		0.43	0.54		0.56	0.59	–	–	
	**FPR**	0.12	0.12		0.12	0.10	0.05	0.04	0.12	0.10		0.05		0.04		0.14	0.12		0.09	0.08	–	–	
**Precipitation (mm) - GLDAS**	**n**	1306			2168		1857		1829			2167				1991			1933		798		
	**R**	0.50	0.68		0.50	0.78	0.24	0.64	0.27	0.61		0.18		0.58		0.34	0.42		0.39	0.67	0.22	0.67	
	**NSE**	0.01	0.20		0.18	0.58	−0.30	0.22	−0.10	0.35		−0.72		−0.61		−0.24	−0.22		0.06	0.44	−0.84	−0.10	
	**MBE**	−1.31	−1.44		−0.13	−0.10	−0.58	−0.57	0.12	0.03		−0.40		−0.42		−2.17	−2.10		0.28	0.27	−0.68	−0.67	
	**RMSE**	12.06	6.13		7.64	2.87	9.71	3.46	9.23	4.12		5.61		2.28		14.07	5.71		7.58	2.86	7.03	2.44	
	**TPR**	0.27	0.28		0.56	0.70	0.33	0.38	0.43	0.47		0.46		0.71		0.32	0.32		0.39	0.43	0.19	0.19	
	**FPR**	0.12	0.11		0.12	0.07	0.13	0.11	0.09	0.06		0.11		0.12		0.15	0.16		0.12	0.10	0.22	0.22	
**Precipitation (mm) - CHIRPS**	**n**	1306			2168		1857		1830			2167				1991			1933		798		
	**R**	0.52	0.74		0.51	0.85	0.27	0.67	0.28	0.69		0.20		0.52		0.35	0.51		0.46	0.73	0.24	0.67	
	**NSE**	0.17	0.50		−0.33	0.49	−0.15	0.36	−0.68	0.27		0.02		0.27		−0.25	0.09		−0.05	0.43	−1.41	−1.07	
	**MBE**	−0.74	−0.90		−0.75	−0.71	−0.41	−0.41	−0.93	−1.07		0.10		0.10		−1.05	−0.94		−0.26	−0.33	−1.69	−1.68	
	**RMSE**	11.07	4.86		9.69	3.17	9.12	3.14	11.42	4.37		4.25		1.54		14.11	4.92		7.98	2.91	8.04	3.34	
	**TPR**	0.27	0.28		0.47	0.81	0.35	0.38	0.38	0.50		0.40		0.68		0.33	0.32		0.30	0.45	0.14	0.19	
	**FPR**	0.12	0.11		0.09	0.04	0.13	0.11	0.11	0.05		0.06		0.12		0.15	0.15		0.07	0.09	0.21	0.23	
**Surface pressure (Pa)**	**n**	1295		1534			–		25				21			2059			2080		–		
	**R**	1.00	0.99	0.92		0.94	–	–	0.95		0.87		1.00		0.96	0.95		0.97	0.99	0.99	–		–
	**NSE**	0.96	0.96	−52.71		−67.65	–	–	−62.80		−70.15		0.99		0.89	−0.47		−1.02	−56.58	−94.83	–		–
	**MBE**	−0.89	−0.90	12.20		12.20	–	–	26.77		26.88		−0.59		−1.33	2.53		2.53	35.87	35.87	–		–
	**RMSE**	1.02	1.07	12.22		12.21	–	–	26.79		26.93		0.99		2.75	2.62		2.57	35.88	35.87	–		–
	**TPR**	0.38	0.37	0.42		0.42	–	–	0.20		0.20		0.20		0.20	0.68		0.71	0.78	0.78	–		–
	**FPR**	0.01	0.01	0.03		0.03	–	–	0.00		0.00		0.00		0.06	0.04		0.03	0.02	0.02	–		–
**Wind speed (m/s)**	**n**	1300		2186			–		2138				2170			2072			2106		–		
	**R**	0.56	0.60	0.89		0.93	–	–	0.25		0.32		0.62		0.71	0.28		0.24	0.48	0.39	–		–
	**NSE**	−7.19	−13.63	0.78		0.84	–	–	−1.32		−1.92		−8.77		−16.73	−0.16		−0.41	−0.49	−1.09	–		–
	**MBE**	−1.89	−1.90	0.04		0.04	–	–	−0.46		−0.46		−1.84		−1.84	0.24		0.24	0.26	0.26	–		–
	**RMSE**	2.08	2.01	0.75		0.58	–	–	0.92		0.76		2.11		2.01	0.58		0.44	0.87	0.52	–		–
	**TPR**	0.26	0.27	0.69		0.72	–	–	0.31		0.36		0.48		0.54	0.32		0.33	0.44	0.35	–		–
	**FPR**	0.10	0.13	0.06		0.06	–	–	0.16		0.14		0.13		0.11	0.15		0.14	0.12	0.15	–		–
**Relative humidity (%)**	**n**	–		–			1854		–				2191			–			1631		–		
	**R**	–	–	–		–	0.79	0.86	–		–		0.68		0.75	–		–	0.87	0.85	–		–
	**NSE**	–	–	–		–	0.40	0.47	–		–		−8.61		−10.48	–		–	0.56	0.37	–		–
	**MBE**	–	–	–		–	4.62	4.61	–		–		33.23		33.24	–		–	−6.34	−6.37	–		–
	**RMSE**	–	–	–		–	9.67	7.92	–		–		34.50		34.01	–		–	10.18	8.97	–		–
	**TPR**	–	–	–		–	0.42	0.48	–		–		0.61		0.70	–		–	0.37	0.36	–		–
	**FPR**	–	–	–		–	0.10	0.07	–		–		0.10		0.08	–		–	0.09	0.09	–		–

a n = number of observations; R = correlation coefficient; NSE = Nash-Sutcliffe efficiency coefficient; MBE = mean bias error; RMSE = Root mean square error; TPR/FPR = True/false positive rate (for days exceeding the 80th percentile); BGD = Dhaka, Bangladesh; BRF = Fortaleza, Brazil; INV = Vellore, India; PKN = Naushero Feroze, Pakistan; PEL = Loreto, Peru; SAV = Venda, South Africa; TZH = Haydom, Tanzania.

**Table 4:** Evaluation statistics for key hydrometeorological variables during peak rotavirus season in the eight MAL-ED sites^a^.

**Variable**		**BGD**		**BRF**		**INV**		**NEB**		**PKN**			**PEL**		**SAV**			**TZH**	
		**Daily**	**7-day average**	**Daily**	**7-day average**	**Daily**	**7-day average**	**Daily**	**7-day average**	**Daily**	**7-day average**	**Daily**		**7-day average**	**Daily**	**7-day average**		**Daily**	**7-day average**
**Peak season**		Nov - Feb		Sep - Nov		Dec - Mar		Dec - Feb		Nov - Feb			Mar - Jun		Feb - Jun			May - Jul	
**Median temperature (C)**	**n**	364		545		622		1756		704			732		877			–	
	**R**	0.89	0.93	0.40	0.56	0.90	0.95	0.95	0.97	0.95	0.98		0.42	0.64	0.88		0.95	–	–
	**NSE**	0.77	0.82	−0.41	−0.22	0.56	0.58	0.55	0.58	0.51	0.57		−2.20	−5.83	0.01		−0.11	–	–
	**MBE**	0.18	0.28	−0.15	−0.15	1.21	1.20	3.30	3.30	−2.32	−2.31		1.48	1.48	3.05		3.05	–	–
	**RMSE**	1.41	1.12	0.71	0.50	1.63	1.37	3.77	3.60	2.63	2.41		1.97	1.62	3.47		3.19	–	–
	**TPR**	0.27	0.28	0.35	0.50	0.59	0.63	0.53	0.56	0.86	0.89		0.39	0.56	0.62		0.69	–	–
	**FPR**	0.09	0.07	0.14	0.12	0.03	0.01	0.10	0.09	0.04	0.02		0.15	0.11	0.08		0.06	–	–
**Precipitation (mm) - GLDAS**	**n**	364		544		626		1487		704			665		817			111	
	**R**	0.50	0.52	0.10	0.16	0.15	0.65	0.27	0.60	−0.01	0.34		0.30	0.34	0.35		0.67	−0.04	−0.06
	**NSE**	0.24	0.23	−1.12	−1.29	−0.21	0.37	−0.10	0.35	−2.63	−2.74		−0.37	−0.40	−0.07		0.43	−18.12	−9.86
	**MBE**	0.06	0.06	−0.01	−0.04	0.21	0.16	0.24	0.16	−0.06	−0.06		−3.13	−2.92	0.07		0.09	−0.42	−0.43
	**RMSE**	2.47	1.55	1.20	0.57	5.91	1.99	10.04	4.49	1.00	0.36		16.27	6.61	5.53		2.18	3.43	1.48
	**TPR**	0.14	0.27	0.24	0.41	0.31	0.39	0.41	0.44	0.07	0.61		0.30	0.31	0.41		0.47	0.16	0.18
	**FPR**	0.11	0.10	0.12	0.14	0.15	0.12	0.09	0.07	0.06	0.14		0.16	0.16	0.12		0.09	0.16	0.27
**Precipitation (mm) - CHIRPS**	**n**	364		544		626		1487		704			665		817			111	
	**R**	0.79	0.74	0.06	0.04	0.16	0.57	0.27	0.68	−0.01	0.12		0.29	0.45	0.39		0.72	0.03	−0.03
	**NSE**	0.55	0.37	−0.12	−0.25	−0.12	0.29	−0.71	0.26	−1.21	−1.33		−0.35	0.04	−0.38		0.44	−15.56	−7.90
	**MBE**	0.07	0.09	0.17	0.16	0.43	0.38	−1.00	−1.19	−0.01	−0.01		−1.29	−1.05	−0.16		−0.22	−0.42	−0.42
	**RMSE**	1.90	1.40	0.87	0.42	5.70	2.11	12.49	4.78	0.78	0.29		16.16	5.49	6.28		2.15	3.19	1.34
	**TPR**	0.05	0.23	0.06	0.21	0.10	0.32	0.38	0.46	0.07	0.39		0.31	0.28	0.25		0.48	0.04	0.18
	**FPR**	0.03	0.16	0.01	0.05	0.05	0.15	0.11	0.06	0.02	0.07		0.15	0.17	0.05		0.10	0.04	0.29
**Peak season**		Nov - Feb		Sep - Nov		Dec - Mar		Dec - Feb		Nov - Feb			Mar - Jun		Feb - Jun			May - Jul	
**Surface pressure (Pa)**	**n**	362		405		–		24		4			669		869			–	
	**R**	0.98	0.95	0.95	0.96	–	–	0.95	0.85	0.97	0.93	0.92		0.95	0.99	0.99		–	–
	**NSE**	0.82	0.67	−56.01	−69.72	–	–	−65.65	−74.68	0.83	0.00	−1.12		−2.48	−67.35	−121.09		–	–
	**MBE**	−0.90	−0.99	12.47	12.44	–	–	26.74	26.85	−1.03	−2.90	2.35		2.35	35.89	35.88		–	–
	**RMSE**	1.02	1.19	12.48	12.45	–	–	26.76	26.90	1.31	3.15	2.45		2.39	35.90	35.88		–	–
	**TPR**	0.32	0.30	0.45	0.43	–	–	0.20	0.20	0.20	0.20	0.59		0.61	0.81	0.82		–	–
	**FPR**	0.03	0.05	0.03	0.04	–	–	0.00	0.00	0.25	0.25	0.06		0.05	0.03	0.02		–	–
**Wind speed (m/s)**	**n**	363		545		–		1756		704			715		877			–	
	**R**	0.50	0.64	0.80	0.81	–	–	0.14	0.16	0.57	0.67	0.35		0.40	0.44	0.32		–	–
	**NSE**	−13.41	−31.06	0.53	0.50	–	–	−1.53	−2.26	−9.08	−21.94	−0.35		−1.03	−0.55	−1.37		–	–
	**MBE**	−1.68	−1.70	0.37	0.36	–	–	−0.43	−0.42	−1.28	−1.27	0.34		0.34	0.35	0.33		–	–
	**RMSE**	1.79	1.74	0.84	0.72	–	–	0.89	0.72	1.42	1.31	0.58		0.45	0.86	0.55		–	–
	**TPR**	0.25	0.28	0.61	0.79	–	–	0.25	0.32	0.56	0.66	0.39		0.33	0.43	0.40		–	–
	**FPR**	0.08	0.08	0.09	0.05	–	–	0.16	0.17	0.10	0.08	0.14		0.15	0.11	0.14		–	–
**Relative humidity (%)**	**n**	–		–		625		–		709			–		625			–	
	**R**	–	–	–	–	0.79	0.83	–	–	0.54	0.66	–		–	0.79	0.72		–	–
	**NSE**	–	–	–	–	0.49	0.55	–	–	−25.69	−45.55	–		–	0.18	−0.24		–	–
	**MBE**	–	–	–	–	2.38	2.42	–	–	37.64	37.72	–		–	−8.49	−8.60		–	–
	**RMSE**	–	–	–	–	7.60	6.16	–	–	38.66	38.17	–		–	11.77	11.05		–	–
	TPR	–	–	–	–	0.41	0.44	–	–	0.46	0.58	–		–	0.33	0.34		–	–
	FPR	–	–	–	–	0.11	0.10	–	–	0.14	0.11	–		–	0.09	0.09		–	–

a n = number of observations; R = correlation coefficient; NSE = Nash-Sutcliffe efficiency coefficient; MBE = mean bias error; RMSE = Root mean square error; TPR/FPR = True/false positive rate (for days exceeding the 80th percentile); BGD = Dhaka, Bangladesh; BRF = Fortaleza, Brazil; INV = Vellore, India; PKN = Naushero Feroze, Pakistan; PEL = Loreto, Peru; SAV = Venda, South Africa; TZH = Haydom, Tanzania.

**Table 5:** Odds ratios (with 95% confidence intervals) for associations between hydrometeorological variables and rotavirus infection adjusting for age, seasonality and calendar time predicted by logistic model fitted with GEE^a^.

		**Daily**			**7-day average**		
		**Station**	**EO-derived**	**Combined**^b^	**Station**	**EO-derived**	**Combined**^**c**^
**Temperature (C)**		1.06***	1.02*	1.02	1.05***	1.02	1.01
		(1.03, 1.09)	(1.00, 1.04)	(1.00, 1.04)	(1.02, 1.09)	(1.00, 1.03)	(0.99, 1.03)
**Precipitation (mm)**	**GLDAS**	1.00	1.02***	1.00	1.02*	1.04***	1.03***
			(1.01, 1.03)	(1.00, 1.01)		(1.03, 1.06)	(1.01, 1.04)
	**CHIRPS**	(0.99, 1.01)	1.01***	1.00	(1.01, 1.04)	1.04***	1.02**
			(1.01, 1.02)	(1.00, 1.01)		(1.02, 1.05)	(1.01, 1.04)
**Surface pressure (mbar)**		1.01***	1.00**	1.00	1.01***	1.00**	1.00
		(1.01, 1.02)	(1.00, 1.00)	(1.00, 1.00)	(1.01, 1.02)	(1.00, 1.00)	(1.00, 1.00)
**Wind speed (m/s)**		0.76***	0.92*	0.95	0.70***	0.87***	0.91**
		(0.69, 0.83)	(0.86, 0.99)	(0.89, 1.01)	(0.62, 0.78)	(0.81, 0.95)	(0.85, 0.97)
**Relative humidity (%)**		0.99	1.01**	1.01*	0.98*	1.01**	1.01**
		(0.98, 1.00)	(1.00, 1.01)	(1.00, 1.01)	(0.96, 1.00)	(1.00, 1.01)	(1.00, 1.02)
**Specific humidity (g/kg)**^c^		–	1.06***	–	–	1.07***	–
			(1.04, 1.09)			(1.04, 1.09)	
**Solar radiation (W/m**^**2**^**)**		–	1.00**	–	–	1.00***	–
			(1.00, 1.00)			(0.99, 1.00)	
**Soil moisture (%)**		–	1.04***	–	–	1.04***	–
			(1.03, 1.05)			(1.03, 1.05)	
**Surface runoff (mm)**		–	1.08***	–	–	1.30***	–
			(1.05, 1.12)			(1.18, 1.43)	

a *** p < 0.001, ** p = 0.001 – 0.01, * p = 0.01 − 0.05. Temperature variables were centered at 25 C; surface pressure at 1000 mbar; wind speed at 2 m/s; relative humidity at 40%; specific humidity at 15 g/kg; solar radiation at 200 W/m^2^; and soil moisture at 25%.

b In the “combined” data, values that were missing in the station data were replaced by their equivalent EO-derived estimates.

c Specific humidity was converted to grams per kilogram (multiplied by 1000) so that the coefficient the change for a plausible one-unit increment.

### 3.1 Temperature

With the exception of the Brazil and Peru sites, there was high correlation (R > 0.85) between the daily temperatures measured at stations and those predicted by the GLDAS model at all sites. While the equivalent correlation estimates were low for Brazil and, especially, Peru, these metrics did improve when temperature was aggregated to 7-day averages (as was the case for all sites). Correspondingly, the Brazil and Peru sites had the lowest level of statistical agreement for temperature according to the NSE statistics, with the negative values for this metric in Loreto, Peru suggesting that the station-based average offers a better estimate. Pakistan was the site with the largest absolute MBE value for temperature and the only one in which the direction of the bias was negative indicating systematic underestimation of the station-based temperature measure by the gridded estimates. The South Africa site, which like the Pakistan site is situated some distance from the weather-station (respectively 36.9 km and 21.9 km), also had high values for MBE and RMSE, but the highest RMSE value for temperature (3.5 °C) was at the Nepal site. At all sites, 7-day temperature averages performed more favorably than daily estimates according to the R, NSE and RMSE, and made only negligible differences to the MBE, with the notable exception of NSE in Peru which deteriorated substantially upon aggregation. Temperature estimates tended to exhibit higher correlation and agreement than other variables but could be biased in either direction by up to 3 degrees. The fact that the lowest correlation coefficients tended to be in Brazil and Peru illustrates the limitation of relying solely on that metric, since these are the two sites that, being closest to the equator, have the least dominant seasonal temperature signal. The TPR for days in the upper quintile was very low and did not improve substantially following averaging over 7-days in all sites, but particularly in Dhaka, Bangladesh and with the exception of Naushero Feroze, Pakistan where around 80% of such extreme-temperature days according to the weather station data where characterized as such by GLDAS. The FPR was highest at the Peru site and lowest at the India site.

Correlation in temperature attenuated only slightly or not at all when data from the off-peak times of the year were excluded at all sites except for in Brazil, where the decrease in this metric was more pronounced, and for 7-day averages in Peru, for which it increased. Similarly, the effect on the NSE for temperature of restricting to rotavirus peak season was mostly slight except in Brazil and for 7-day averages in South Africa, where it changed qualitatively from a positive to a negative value. The MBE and RMSE for temperature increased only slightly in most cases, but in Brazil the direction of the bias changed, while the effect on the TPR and FPR was inconsistent across sites, improving most in Pakistan, deteriorating in Brazil and changing very little in Bangladesh.

A 1 degree increase in daily temperature was highly statistically significantly associated with a 6% increase in the odds of rotavirus detection when measured at the weather stations, but only a slightly statistically significant 2% increase predicted by the EO estimates, which was no longer significant when the combined data were used. When the same models were fitted using the 7-day average temperatures, the EO effect estimate lost significance while the station data effect attenuated slightly but retained its high level of significance. Sites that were in similar climatic zones – Bangladesh and India, and Nepal and South Africa – tended to be similar with respect to their evaluation statistics for temperature.

### 3.2 Precipitation

The distribution of the precipitation parameter is highly skewed to the right with a high proportion of days with zero rainfall at all sites and for all data sources, but particularly in Pakistan and at the two African sites. Both the GLDAS and the CHIRPS precipitation estimates were poorly correlated (R < 0.55) with the station-based daily volumes at all sites. When the precipitation variables were aggregated into 7-day averages, the correlation improved at all sites with CHIRPS tending to outperform GLDAS, exceeding R = 0.6 in all but Peru and Pakistan. In several sites, the two products were biased in opposite directions, and in no site did one outperform the other across all evaluation statistics. Notably, there also seemed to be no clear relation between a site's distance from its weather-station and the performance of its precipitation estimate. Both GLDAS and CHIRPS had very low sensitivity (TPR) for classifying extreme precipitation days, although for both products, this metric improved greatly in Brazil and Pakistan for 7-day average precipitation. By far the highest FPRs were seen for the Tanzania site for both products.

For the precipitation variables, there was considerable variation in how the evaluation metrics changed when the analysis focused on the peak rotavirus season depending on site, period of aggregation and, to some extent, source (i.e. GLDAS compared to CHIRPS). At the Nepal, Peru and South Africa sites the differences were slight across all metrics for both sources, while in the other sites, but most markedly, in Tanzania, there was an apparent tendency for correlation and NSE to decrease substantially, while MBE and RMSE reduced slightly. The TPR tended to decline for the peak rotavirus period compared with the full dataset, while the high FPR for the Tanzania site decreased for the daily averages (most markedly with CHIRPS), but increased slightly for the 7-day averages.

No statistically significant association was found between daily precipitation measured by weather stations and rotavirus positivity, though a slightly statistically significant association was observed when the variable was averaged over 7 days. By contrast, the GLDAS and CHIRPS estimates respectively predicted a highly statistically significant 2% and 1% increase in the odds of a rotavirus positive stool for every millimeter increase in daily precipitation, and a much larger and similarly significant effect size when averaged over 7 days. Substituting missing precipitations data from stations with EO-estimates did not improve the ability of the model to detect a statistically significant effect compared to the station-only model when a daily resolution was considered, however, this combined variable showed respectively a highly and a moderately statistically significant association when averaged over seven days using GLDAS and CHIRPS.

### 3.3 Surface pressure

GLDAS surface pressure estimates were highly correlated with station-based estimates (R > 0.85), even at those sites with very few such observations, namely Nepal (n = 25) and Pakistan (n = 21). Performance according to the other metrics was much more varied, with the Brazil, South Africa and Nepal sites showing a very clear systematic bias towards over-estimation of the station-based measures by the gridded estimates and poor statistical agreement between the two sources according to the NSE. The high RMSE at these sites were in part due to the fact that this statistic is expressed in the same units as the variable itself, in this case millibars with values at a higher order of magnitude than the other variables. The TPR tended to be low for both daily and 7-day surface pressure estimates, particularly at the sites with very few observations, but with the exception of Peru and, especially, South Africa. The FPR for surface pressure was low at all sites compared with other parameters. In general, the evaluation statistics for surface pressure deteriorated when only the peak season was considered, but not substantially. Only a very small number of pressure observations from the Pakistan site occurred in the peak rotavirus season. For both daily and 7-day average estimates, a one millibar increase in surface pressure above 1000 mbar was associated with a highly statistically significant 1% increase in the odds of rotavirus detection when weather station records were used, a moderately statistically significant < 0.5% increase when GLDAS was used and had no statistically significant effect when the combined data was used.

### 3.4 Wind speed

The wind speed estimated by GLDAS tended to show poor correlation with those recorded by the weather stations and exhibit considerable biases and poor statistical agreement. An exception to this was the Brazil site, where the EO-derived wind speed estimate was notable for showing exceptionally high correlation and agreement and minimal bias. The TPR for wind speed estimated by GLDAS was low and the FPR high relative to other parameters, again with the exception of the Brazil site. A highly statistically significant inverse association between weather station measurements of wind speed and the outcome was observed with a 1 m/s increase in daily wind speed predicting a 24% decline and 7-day average wind speed a 30% decline in the odds of rotavirus infection. The magnitude and the statistical significance of the effect attenuated substantially when GLDAS daily estimates were used instead, although 7-day averages of the EO variable did obtain a similarly high level of statistical significant, but for an effect of lower magnitude than the station-based observations.

### 3.5 Relative humidity

Relative humidity showed moderate correlation between the GLDAS and station-based data in South Africa and, at least considering the 7-day average, in India, showing moderate statistical agreement by the NSE, but with notable error and bias in opposite directions in the two sites. In Pakistan, the equivalent estimates were only moderately correlated and showed low agreement and high bias and error. It should be noted in interpreting these statistics, that the station-based estimates for Pakistan and India were calculated as the average of two daily measurements taken at time points representing extremes of the daily cycle of humidity, while the GLDAS indicator was an average of 3-hourly estimates within a day, a fact which may explain some of the bias and error seen at these sites. Correlation between the EO-derived and weather station estimates of relative humidity from all three sites either decreased slightly or did not change when only the peak rotavirus season was considered. At the India site NSE increased slightly while MBE and RMSE decreased while in the other two sites with relative humidity data, the opposite was the case. Weather station records of relative humidity were not statistically significantly associated with rotavirus at a daily resolution and only slightly so when aggregated to 7-day means, however the EO-derived estimates were moderately statistically significant at both levels and the combined data was slightly statically significantly associated with the outcome when daily estimates were considered and moderately so for 7-day averages.

### 3.6 Other parameters

Of the four GLDAS variables for which no station-based equivalents were available (specific humidity, solar radiation, soil moisture and surface runoff), all showed a highly statistically significantly direct association with rotavirus infection for both daily and 7-day average estimates with the exception of solar radiation, for which the association was inverse (not apparent from Table 5 due to rounding), and moderately statistically significant for daily estimates.

## 4 Discussion

The increased availability of historic meteorological data offers great potential to environmental epidemiologists that has yet to be fully explored. While weather stations may record a small number of parameters at particular strategic locations to varying degrees of accuracy, EO-derived products aim to provide meteorological estimates where direct measurements do not exist and therefore merit assessment as potential surrogates. Although such data are starting to be used in studies of human health, livelihood and vulnerability (Grace et al., 2015, Moore et al., 2017, Jagai et al., 2012, López-Carr et al., 2014), as yet there have been no systematic attempts to evaluate the relative validity and utility of hydrometeorological data from different sources for modeling health outcomes. This study represents an initial attempt to do this and the results indicate there may be certain pitfalls to straightforwardly substituting ground-based observations for their EO-derived equivalents and that researchers should be cautious about the unreflective reliance on these without proper consideration of their limitations.

According to the evaluation statistics the performance of the two gridded EO data products assessed here was highly dependent on the location, the variable, the evaluation metric and the distance from the study site (the location at which the data were extracted from the grid) to the weather station at which the in situ data were recorded. Furthermore, several variables differed considerably in their ability to statistically significantly predict rotavirus infection depending on whether the station-based or EO-derived data were used and, when the latter was used to fill gaps in the former, it often led to a considerable attenuation of the significance level. Temperature estimates from GLDAS were one of the best performing variables according to the evaluation statistics, yet showed at best only a slightly statistically significant association with the rotavirus outcome, while their station-based equivalents showed a considerably higher level of statistical significance, despite their incompleteness. Estimates of precipitation performed most favorably according to the evaluation statistics when they were extracted from CHIRPS rather than GLDAS and were aggregated to 7-day averages. In absolute terms, however, precipitation was one of the variables for which EO-derived data performed the worst. This is perhaps unsurprising, given that it is a challenging variable both to measure remotely and to model, since variation in rainfall can be so localized as to confound simple grid-to-station comparisons, especially at the comparatively low resolution of CHIRPS and GLDAS. In spite of this, EO-derived precipitation estimates showed strongly statistically significant associations with rotavirus where gauge-based estimates showed no or only weak associations, particularly for GLDAS but also for CHIRPS. This may be because the model-derived estimates are not in fact reflective of rainfall per se, but are some aggregate of closely related factors like humidity, cloud and wind which correlate with precipitation at large scale, but differ from true precipitation in subtle ways that collectively make them a stronger driver of rotavirus transmission. It is also conceivable that EO actually provide more meaningful rainfall estimates than station data in some cases, due to station equipment malfunction or siting bias. This is difficult to evaluate with available data.

Daily surface pressure from GLDAS was the variable that showed the highest level of correlation with the station-based measures both for the full annual cycle and for the peak-season, including in Brazil and Peru which, as with temperature, had the smallest seasonal variation. The biases observed for this variable are consistent with differences in altitude between the sites and their respective weather stations. Confirming the findings of Hervas and colleagues (Hervás et al., 2014), pressure was statistically significantly associated with rotavirus. This was most marked for weather station data and slightly less so for EO data, but no longer held true when the two were used in combination. That wind speed mostly performed poorly is largely to be expected since most weather stations only report winds at a 2 m height on a very localized scale, while GLDAS produces broader scale estimates of 10 m height winds. This may also explain why station-based wind measurement were strongly associated with rotavirus, while daily EO-derived estimates showed a weaker association. Wind speeds at 2 m are more likely to facilitate the transmission of the virus than at 10 m. That the station-based measurements of surface pressure and wind speed were so highly statistically significant in spite of their incompleteness is suggestive of a strong and hitherto underexplored association (Hervás et al., 2014, Levy et al., 2009). In an analysis that had used only EO-derived estimates of wind speed, this association would have appeared much less striking, illustrating that a poorly informed choice of meteorological data can be a potential source of type II error.

In line with previously documented evidence (D’Souza et al., 2008, Levy et al., 2009, Hashizume et al., 2008), an association was found between relative humidity and rotavirus over the 3-day lag used here, however, it was one of the weaker associations identified and only apparent when EO-derived or combined estimates were used or, to a lesser extent, when station measurements were averaged over seven days. It should be noted that the choice to examine the association over a 3-day lag, though guided by biology, was to some extent arbitrary. Further exploration of the exposure-lag-response structure may reveal a stronger association operating over longer time windows but is beyond the scope of this paper (Gasparrini, 2014). It is notable that the two measures of humidity differed substantially in their association with rotavirus, indicating a highly statistically significant association with specific humidity but a more moderate one for relative humidity. This demonstrates the importance of considering the physical meaning of related but distinct variables: specific humidity is highly sensitive to air temperature, and thus reflects a combination of temperature and humidity conditions, where relative humidity is standardized to temperature and represents degree of saturation.

Many previously published analyses of the influence of weather on rotavirus or other health outcomes have tended to aggregate the meteorological exposures over large areas or longer time windows (e.g. weeks or months). This study demonstrates for the first time that associations can still be detected using daily estimates, which in many cases were more highly statistically significantly associated with the outcome than 7-day averages. Where outcome data is available with the precise date of ascertainment, an equally high resolution for the exposure data may be preferred in order to retain the variability in the data and for the most precise characterization of lag effects and the temporal order over which multivariate associations operate. In this preliminary analysis we assumed linearity in all associations between hydrometeorological parameters and rotavirus. Future analyses should include methods capable of taking into account non-linear relationships such as polynomial transformations or natural cubic splines.

With a few notable site-specific exceptions, the EO data performed very poorly in detecting extremes in the weather station data, which here we defined as sensitivity in classifying days in which a given parameter exceeded the 80th percentile of its overall distribution. Researchers wishing to assess the impact of extreme weather events on health outcomes, are encouraged to explore multiple cutoffs and definitions, as well as lower extremes, which were beyond the scope of this paper, but may be particularly relevant for parameters such as wind speed given its inverse association with rotavirus identified here.

That the global models and EO data used to generate the variables included here do not perform perfectly should not be surprising. Neither GLDAS nor CHIRPS purport to be entirely representative at local scale or daily resolution, however they do offer the advantage that findings can be generalized to other locations and results mapped continuously across the landscape. While there is no *a priori* reason to suppose that one EO product is better than another, GLDAS and CHIRPS were chosen for this analysis because they are two products for which promising validation work has been published. There are numerous initiatives underway to evaluate these and similar datasets in a more robust way across multiple locations, however, to date, most validation efforts have been piecemeal and the reality remains that in most locations, like those of the MAL-ED study sites, the data remain unevaluated. Spot-check comparisons like the one reported in this study often yield conflicting or inconclusive conclusions, since the station-based data do not always represent a gold standard of comparison for estimates extracted from gridded products at precise coordinates, especially for parameters like precipitation that vary on such local scales. Different weather stations may use different equipment to measure the same parameter and, because of this and other factors, may vary widely in their accuracy in characterizing conditions at their own locations and in the extent to which such characterizations can be extrapolated to nearby population settlements. When, as is the case with this study, there is disagreement between the two data sources, it is near impossible to attribute this to specific sources of error such as deficiencies with the model used to derive the EO estimate, distance between the study site and its nearest weather station or incompleteness or inaccuracies in the station records. Poor performance of data from one source relative to the other is problematic insofar as it impedes the ability of a study to detect an association and may be tolerated to the extent that it is still possible to detect and quantify their effect on outcomes that there is *a priori* reason to believe are climate-sensitive.

As weather stations become more affordable, accurate, easy to install and offer a wider suite of measurements, environmental epidemiologists working in remote and underserved field sites should consider installing these instruments themselves. Otherwise, secondary station-based observation data should be given preference when they are complete and measured at a location that is close to the study site, and exhaustive attempts should be made to coordinate with local agencies that might be able to provide such data when they are not publicly accessible. When, as is often the case, high quality observational data are not available, EO-derived products may be introduced in a number of ways: to fill gaps, either by direct substitution, or as covariates for multiple imputation of missing data; as surrogates for variables that are not commonly measured at weather stations (e.g. soil moisture, surface runoff etc.); to generate data ensembles by averaging over multiple EO-derived data sets (the “wisdom of crowds” approach); to set uncertainty bounds when applying data to health risk assessment.

Researchers may feel justified in using gridded products as surrogates to the extent that they are the best hydrometeorological monitoring tools available at global scale and daily resolution. Alternatively, they may use observed data to calibrate and adjust the gridded estimates if they have a level of confidence that the station-based records truly represent the historical conditions at their sites, or attempt custom corrections based on characteristics of the study area, where these are known to high degree of certainty. Where observational data is available but incomplete, studies should report associations with the observed as well as EO-derived data as a sensitivity analysis. The nature of the research question will, in some respects, determine the relative importance of the different evaluation metrics. If the absolute values of the hydrometeorological variable are of interest, minimizing bias will be a priority, whereas if climate anomalies relative to the normal range are the predictor of interest, then more bias may be tolerated. Several of the commonly used evaluation metrics may be sensitive to the averaging period and, as demonstrated here, to the seasonal cycle. The performance of peak-season data would be most important when developing predictive models intended to predict more than just the seasonality of a disease process. However, what is significant from the point of view of the data's epidemiological application is how sensitive the analysis is to moderate inaccuracies in the weather data.

## 5 Conclusions

In this study, standard metrics were applied to a set of ten hydrometeorological variables extracted from two gridded climate data products to evaluate their performance relative to weather station-derived estimates at eight specific geographic locations. The performance of these estimates varied considerably within the same location, for the same variable across locations, according to different evaluation criteria and for the peak season compared to the full dataset in ways that showed no obvious pattern. Later these variables were each used in longitudinal regression models to test their association with rotavirus infection and again, the results were found to vary with neither data source outperforming the other across all variables. For some variables, the station-based records showed a strong association while the equivalent EO-derived estimates showed a much weaker one, while for others, the opposite was true. These results should serve as a reminder to researchers wishing to utilize climate data sets to recognize both in the analysis and the interpretation of the results that EO-derived data essentially amount to estimates that have rarely been validated in locations like the MAL-ED study communities or those that might commonly be selected for community-based epidemiological studies. This analysis constitutes a spot test at eight locations, but one that is indicative of climate conditions and data quality and availability in many locations. The question of which data source is most suitable will depend on the particular application to health data. Where feasible, epidemiologists engaged in prospective research into environmentally driven diseases should install their own weather monitoring stations at their study sites, in order to circumvent the constraints of choosing between distant or incomplete station data or unverified EO estimates.

## Footnotes

1 Field calibration against references for 0 – 90% RH. For 90–100% RH, ± 3%, and against factory references, ± 1% (Vaisala, 2006).

2 11 observations, < 0.0001% of the overall data.
