# Supplementary material for: Evaluating meteorological data from weather stations, and from satellites and global models for a multi-site epidemiological study
Source: Environ Res. 2018 Aug;165:91–109. doi: 10.1016/j.envres.2018.02.027 (PMC6024078; doi:10.1016/j.envres.2018.02.027)
Supplement: Supplementary file 1 — Supplementary material [file mmc1.docx]

## **Supplementary Material**


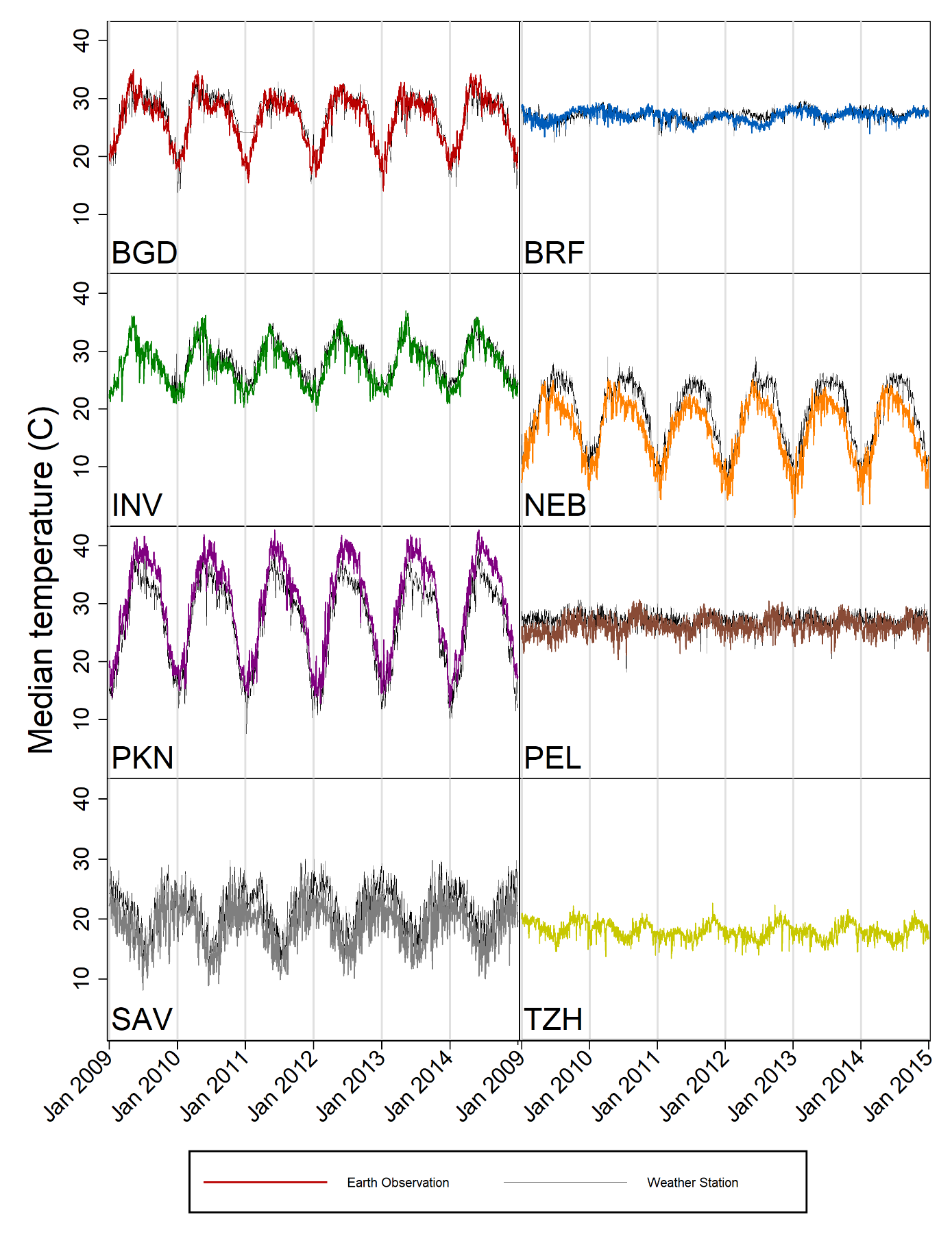
Supplementary figure 1: Daily median temperature (C) estimates by MAL-ED site, 2009 - 2014


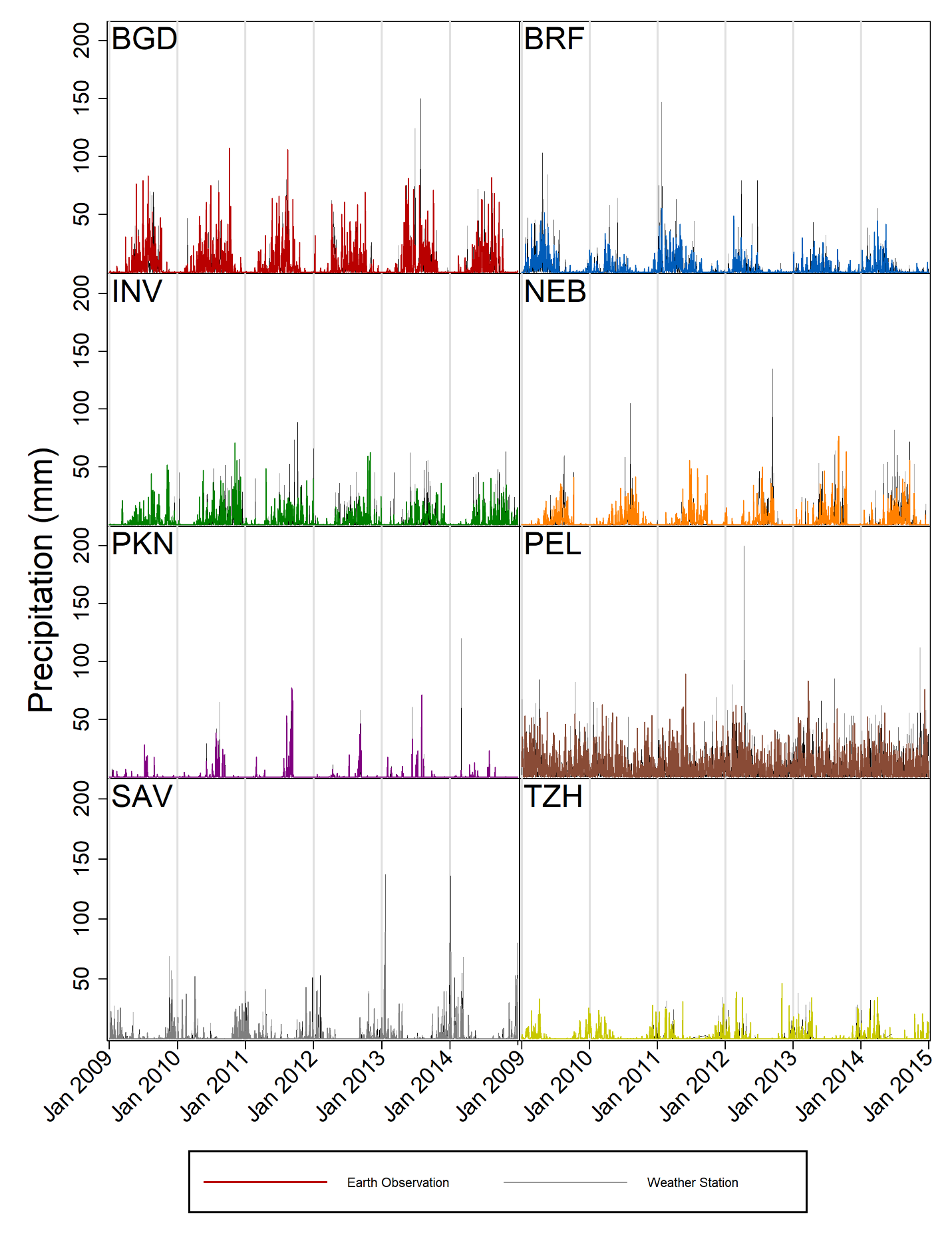


Supplementary figure 2: Daily precipitation (GLDAS - mm) estimates by MAL-ED site, 2009 - 2014


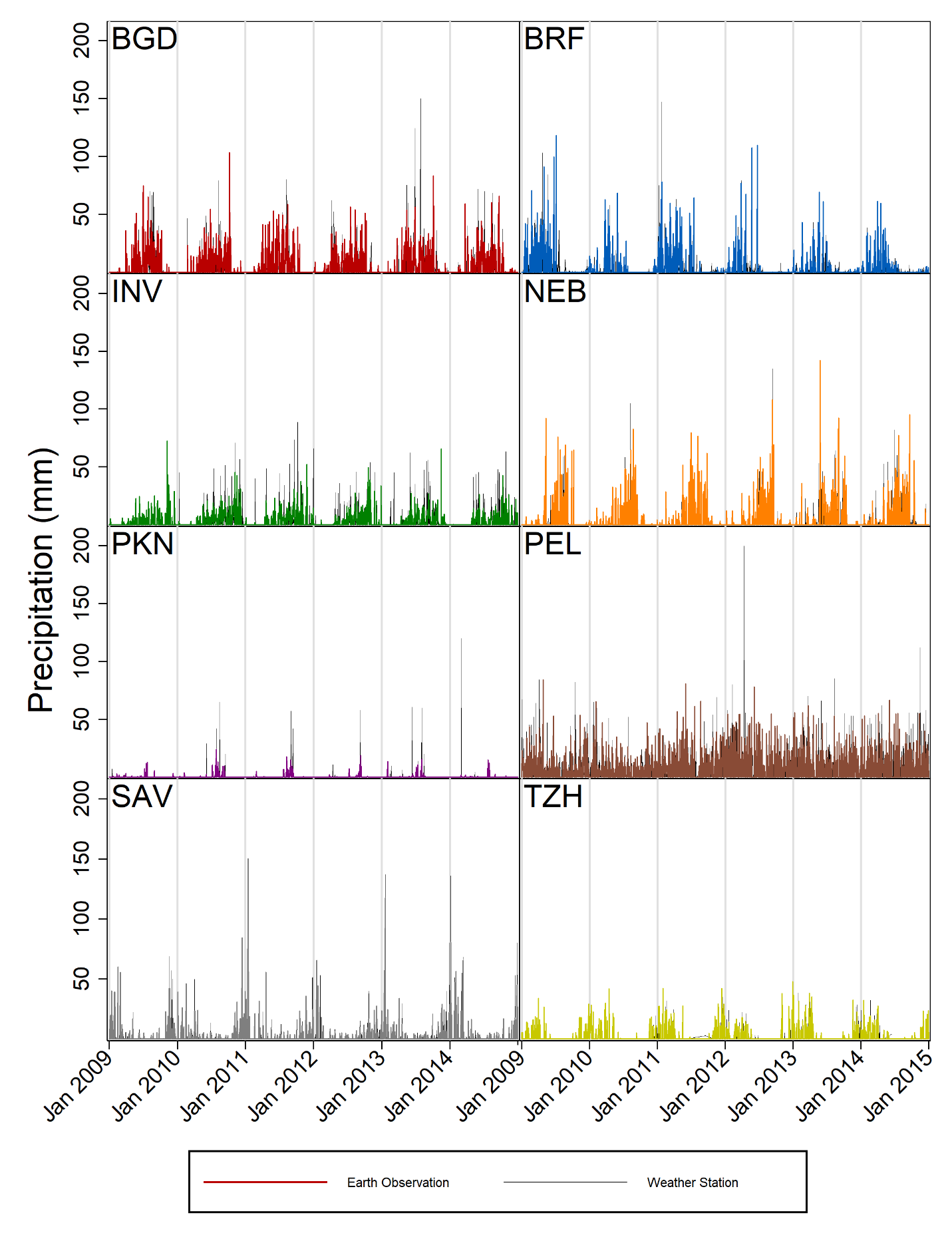


Supplementary figure 3: Daily precipitation (CHIRPS - mm) estimates by MAL-ED site, 2009 - 2014
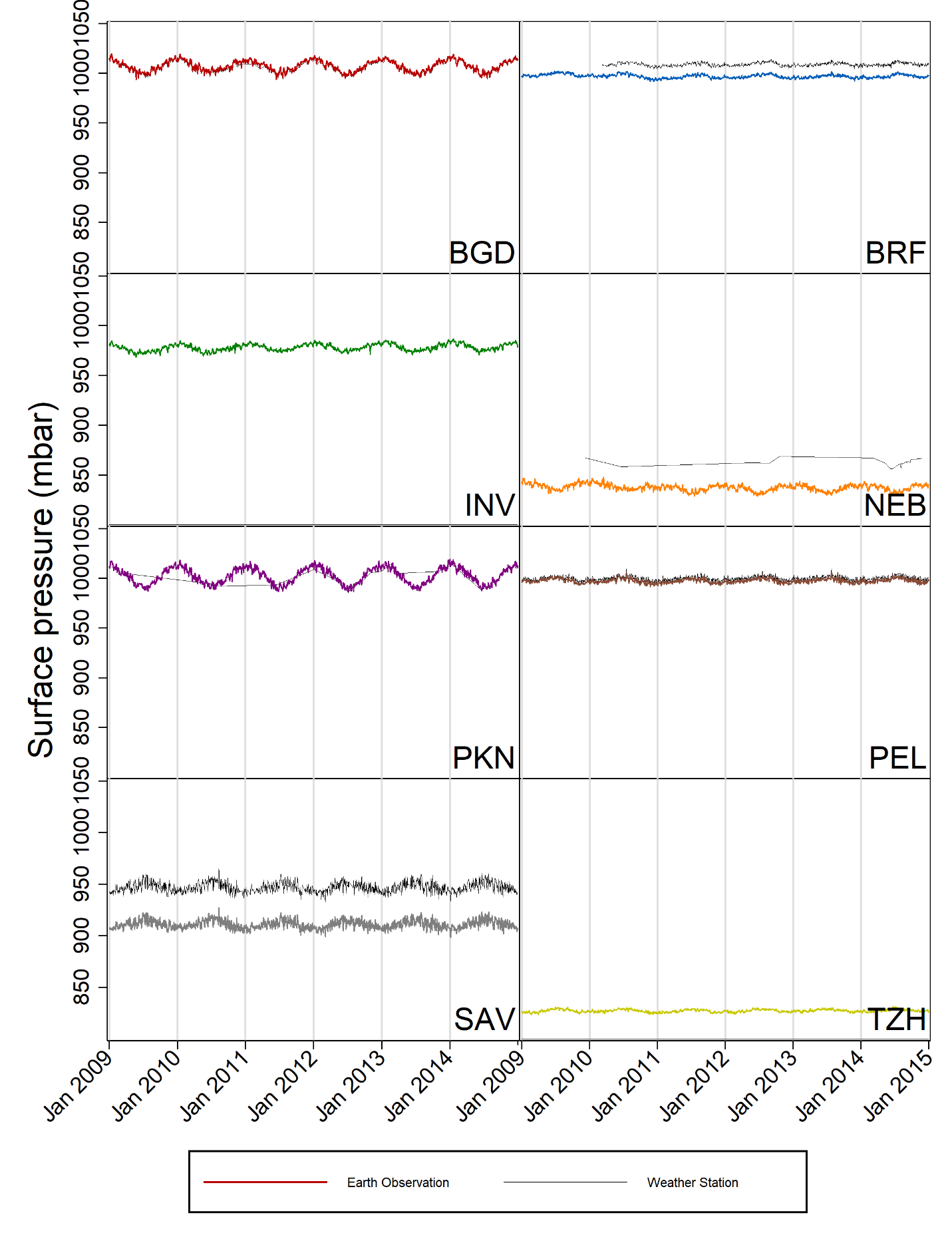


Supplementary figure 4: Daily surface pressure (mbar) estimates by MAL-ED site, 2009 – 2014 (with an offset applied to correct for a temporal discontinuity in Tanzania)
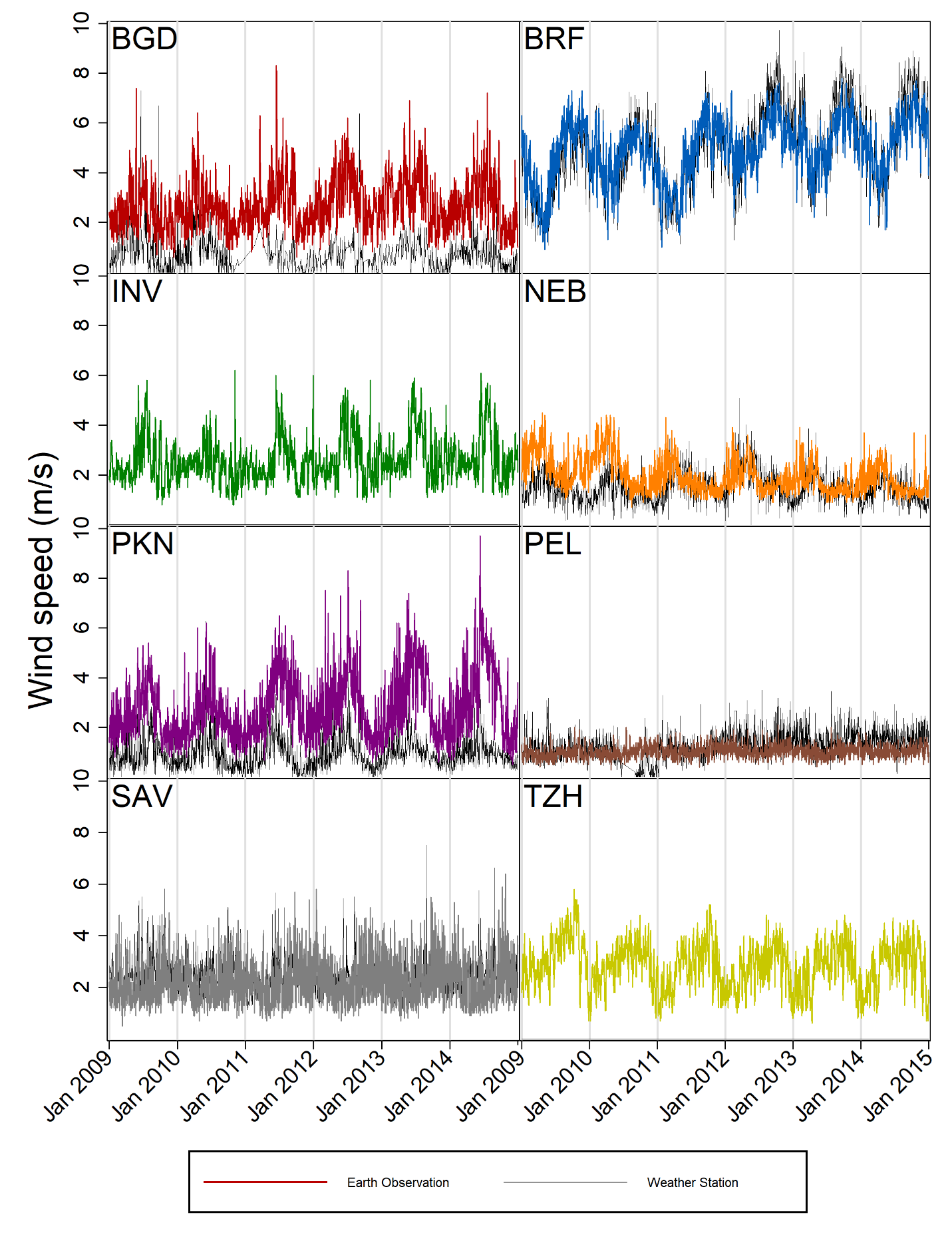


Supplementary figure 5: Daily wind speed (m/s) estimates by MAL-ED site, 2009 - 2014


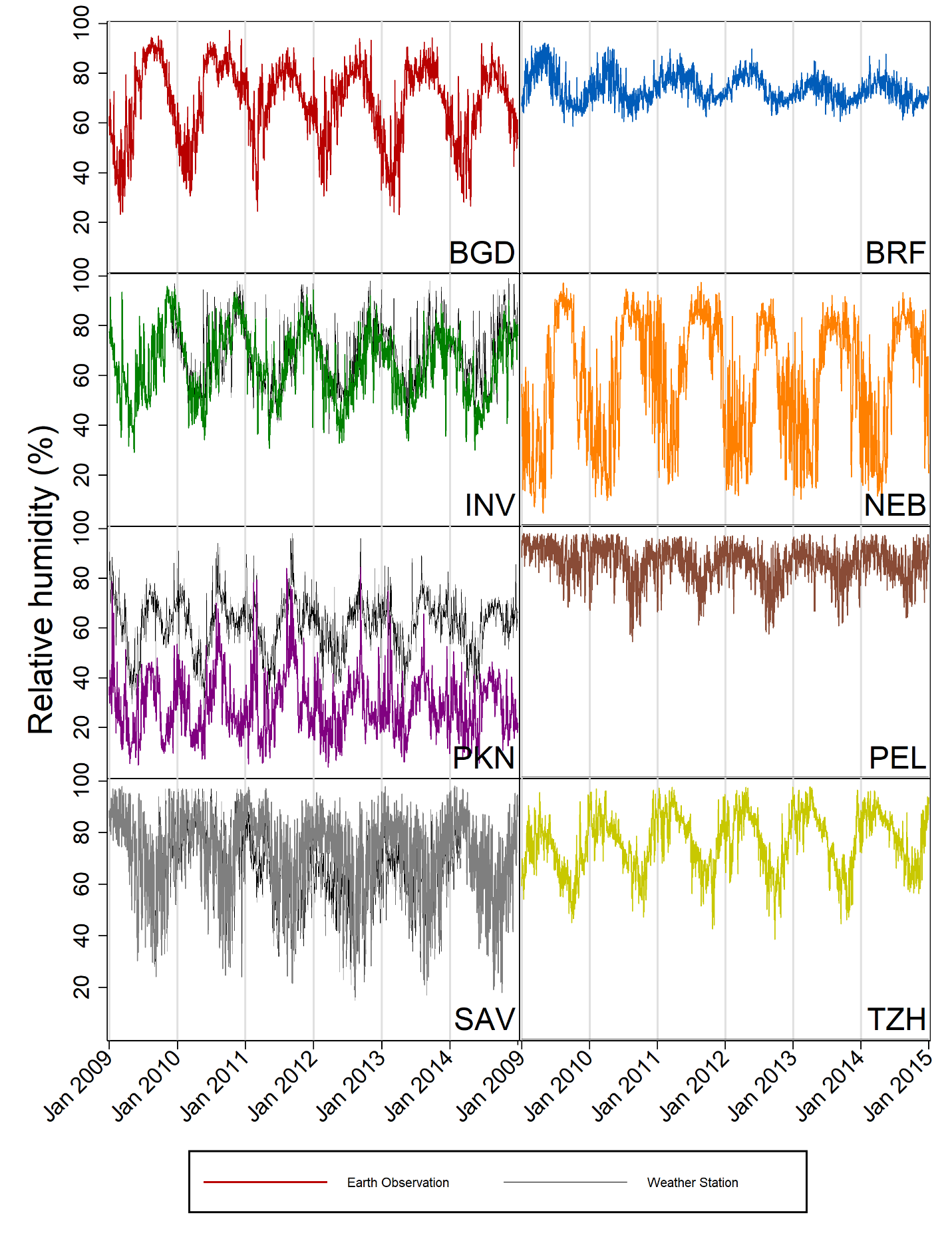


Supplementary figure 6: Daily relative humidity (%) estimates by MAL-ED site, 2009 - 2014


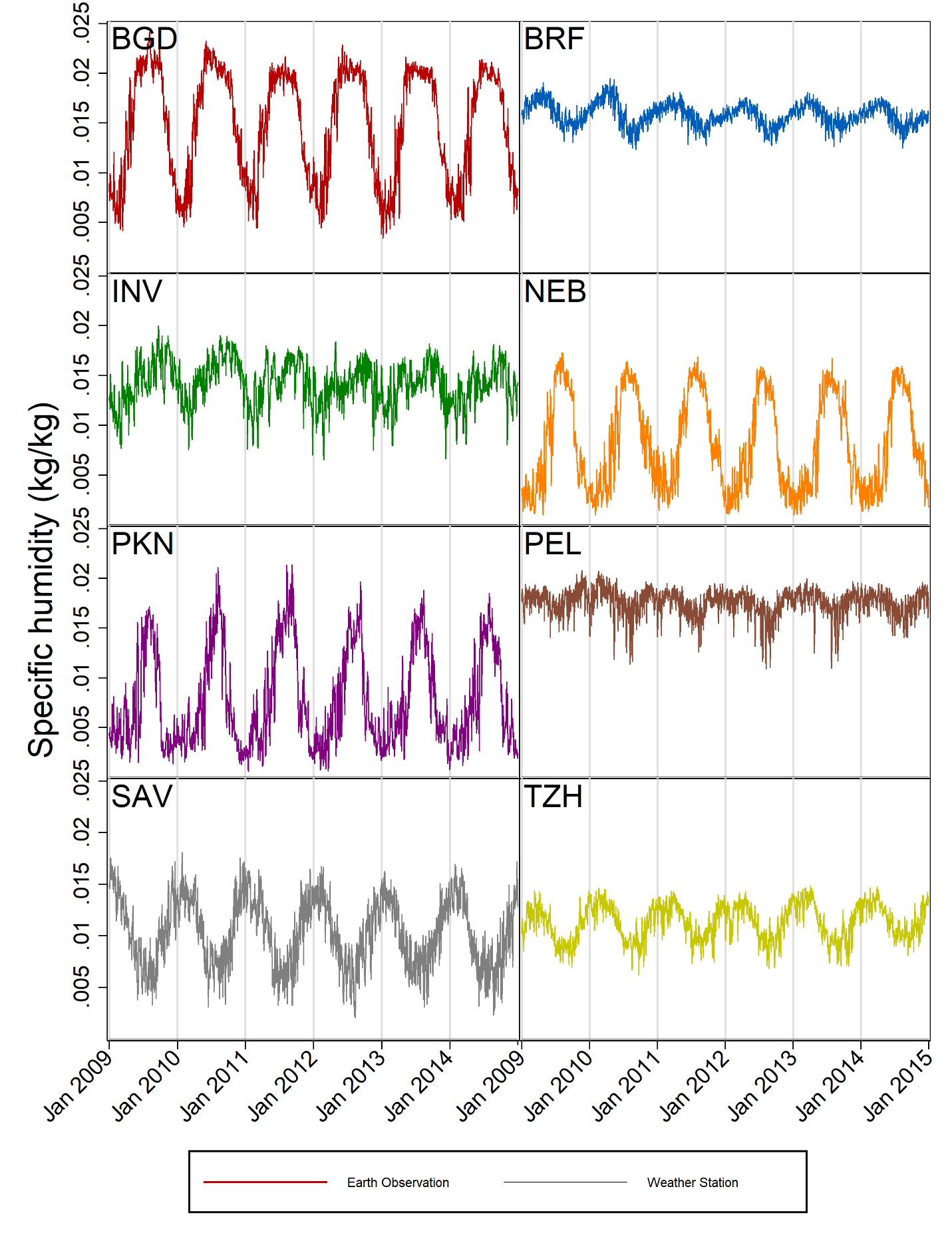


Supplementary figure 7: Daily specific humidity (kg/kg) estimates by MAL-ED site, 2009 - 2014


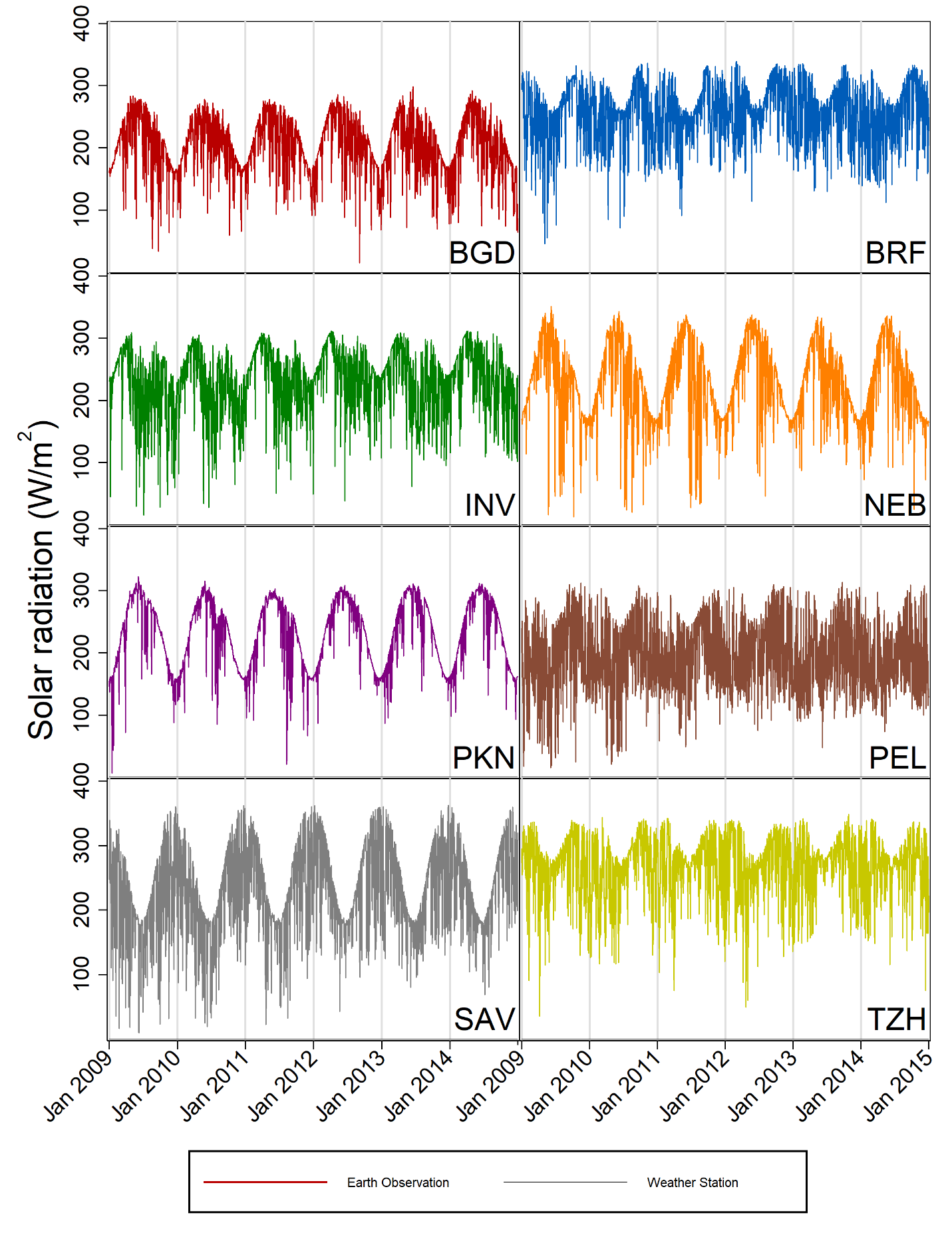
 Supplementary figure 8: Daily solar radiation (W/m2) estimates by MAL-ED site, 2009 - 2014


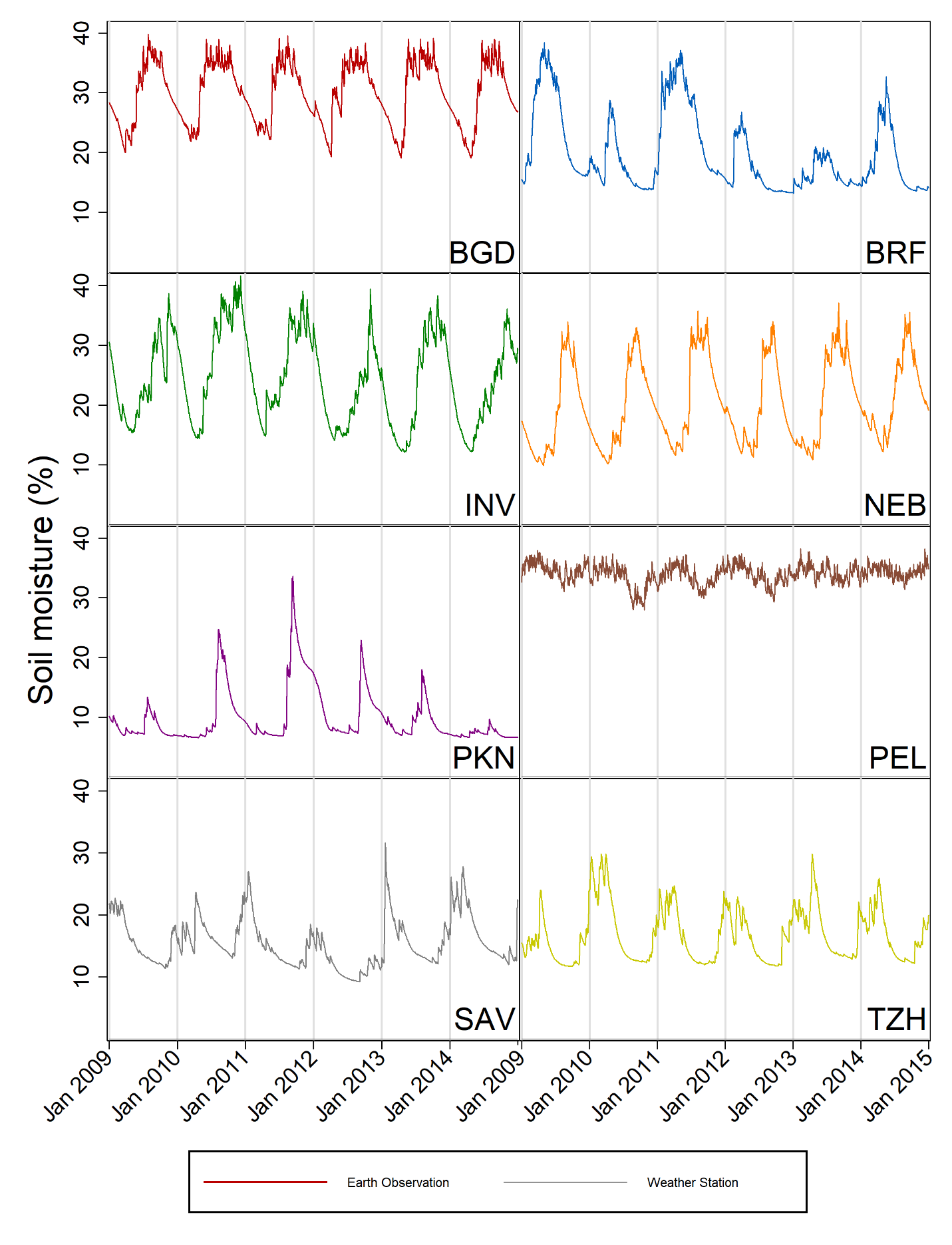


Supplementary figure 9: Daily soil moisture (%) estimates by MAL-ED site, 2009 - 2014


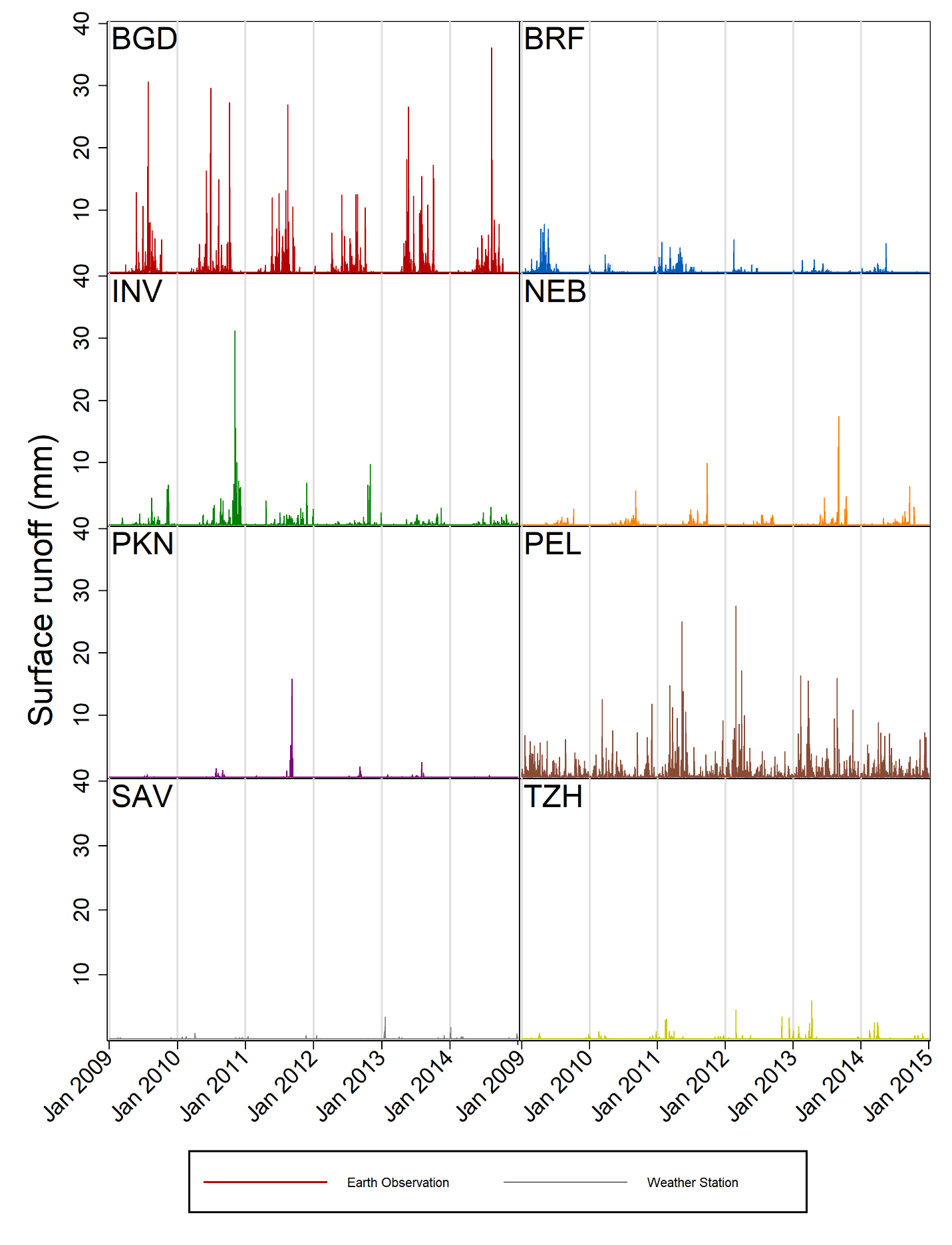


Supplementary figure 10: Daily surface runoff (mm) estimates by MAL-ED site, 2009 – 2014

| Table S1: Summary of GLDAS- CHIRPS and weather-station-based hydrometeorological variables from the closest weather stations to each of the eight MAL-ED sites | | | | | | | | | | | |
| --- | --- | --- | --- | --- | --- | --- | --- | --- | --- | --- | --- |
| Variable | | **Median temperature (C)** | | **Precipitation (mm)** | | | | **Surface pressure (mbar)** | | **Wind speed (m/s)** | |
|  |  | **GLDAS** | **Station** | **GLDAS** | **CHIRPS** | **Station** | | **GLDAS** | **Station** | **GLDAS** | **Station** |
| Bangladesh | Median | 27.9 | 28.8 | 0.0 | 0.0 | 0.0 | 1,007.3 | | 1,005.7 | 2.6 | 0.6 |
|  | IQR | 6.3 | 5.5 | 4.3 | 6.1 | 1.8 | 8.9 | | 8.6 | 1.4 | 0.8 |
|  | Maximum | 35.0 | 33.9 | 107.1 | 104.3 | 150.1 | 1,019.6 | | 1,018.3 | 8.3 | 7.3 |
|  | Minimum | 14.1 | 13.8 | 0.0 | 0.0 | 0.0 | 993.5 | | 992.8 | 0.6 | 0.0 |
|  | Completeness | 100.0% | 59.7% | 100.0% | 100.0% | 59.6% | 100.0% | | 59.1% | 100.0% | 59.3% |
| Brazil | Median | 27.1 | 27.2 | 0.0 | 0.0 | 0.0 | 996.9 | | 1,008.7 | 5.0 | 4.9 |
|  | IQR | 1.4 | 1.3 | 1.7 | 0.0 | 1.0 | 2.7 | | 2.3 | 1.7 | 2.3 |
|  | Maximum | 29.4 | 29.8 | 55.0 | 109.6 | 147.1 | 1,002.2 | | 1,014.0 | 7.8 | 9.7 |
|  | Minimum | 23.3 | 22.5 | 0.0 | 0.0 | 0.0 | 991.5 | | 1,003.8 | 0.9 | 1.1 |
|  | Completeness | 100.0% | 99.8% | 100.0% | 100.0% | 99.0% | 100.0% | | 70.0% | 100.0% | 99.8% |
| India | Median | 28.0 | 28.9 | 0.0 | 0.0 | 0.0 | 978.0 | | - | 2.4 | - |
|  | IQR | 5.8 | 5.1 | 2.9 | 3.6 | 0.2 | 5.6 | | - | 1.0 | - |
|  | Maximum | 37.0 | 36.5 | 70.7 | 75.6 | 88.4 | 986.8 | | - | 6.2 | - |
|  | Minimum | 19.6 | 20.9 | 0.0 | 0.0 | 0.0 | 968.4 | | - | 0.8 | - |
|  | Completeness | 100.0% | 84.5% | 100.0% | 100.0% | 84.8% | 100.0% | | - | 100.0% | - |
| Nepal | Median | 17.8 | 20.5 | 0.0 | 0.0 | 0.0 | 837.6 | | 862.1 | 1.8 | 1.4 |
|  | IQR | 8.5 | 9.5 | 2.1 | 0.0 | 0.0 | 4.7 | | 2.5 | 1.0 | 0.8 |
|  | Maximum | 25.2 | 29.0 | 76.5 | 125.7 | 134.6 | 847.8 | | 868.7 | 4.5 | 5.1 |
|  | Minimum | 1.3 | 5.5 | 0.0 | 0.0 | 0.0 | 828.7 | | 856.1 | 0.7 | 0.0 |
|  | Completeness | 100.0% | 97.6% | 100.0% | 100.0% | 83.5% | 100.0% | | 1.1% | 100.0% | 97.6% |
| Pakistan | Median | 31.7 | 29.2 | 0.0 | 0.0 | 0.0 | 1,001.3 | | 994.8 | 2.6 | 0.9 |
|  | IQR | 13.8 | 12.8 | 0.0 | 0.0 | 0.0 | 13.8 | | 12.7 | 2.0 | 0.8 |
|  | Maximum | 42.5 | 40.5 | 76.9 | 30.3 | 119.9 | 1,017.5 | | 1,015.6 | 10.5 | 6.3 |
|  | Minimum | 12.3 | 7.5 | 0.0 | 0.0 | 0.0 | 984.4 | | 985.2 | 0.7 | 0.0 |
|  | Completeness | 100.0% | 99.0% | 100.0% | 100.0% | 98.9% | 100.0% | | 1.0% | 100.0% | 99.0% |
| Peru | Median | 26.2 | 27.4 | 4.4 | 0.0 | 0.8 | 997.2 | | 999.8 | 1.0 | 1.2 |
|  | IQR | 1.9 | 1.4 | 11.5 | 11.2 | 7.4 | 3.1 | | 3.0 | 0.3 | 0.7 |
|  | Maximum | 30.7 | 30.6 | 88.9 | 84.1 | 199.9 | 1,005.4 | | 1,009.4 | 2.1 | 3.5 |
|  | Minimum | 20.4 | 18.1 | 0.0 | 0.0 | 0.0 | 991.1 | | 993.1 | 0.3 | 0.0 |
|  | Completeness | 100.0% | 100.0% | 100.0% | 100.0% | 90.9% | 100.0% | | 94.0% | 100.0% | 94.6% |
| South Africa | Median | 21.4 | 21.9 | 0.0 | 0.0 | 0.0 | 946.8 | | 946.5 | 2.0 | 2.4 |
|  | IQR | 5.5 | 5.8 | 0.0 | 0.0 | 0.3 | 7.1 | | 6.8 | 1.1 | 0.8 |
|  | Maximum | 32.0 | 29.9 | 91.3 | 116.7 | 136.9 | 967.1 | | 965.0 | 6.1 | 7.5 |
|  | Minimum | 10.5 | 9.9 | 0.0 | 0.0 | 0.0 | 934.1 | | 932.7 | 0.5 | 1.0 |
|  | Completeness | 100.0% | 96.1% | 100.0% | 100.0% | 88.2% | 100.0% | | 94.9% | 100.0% | 96.1% |
| Tanzania | Median | 18.7 | - | 0.0 | 0.0 | 0.0 | 831.5 | | - | 3.0 | - |
|  | IQR | 1.9 | - | 0.3 | 0.0 | 0.0 | 2.1 | | - | 1.3 | - |
|  | Maximum | 22.8 | - | 46.5 | 63.1 | 38.5 | 836.1 | | - | 5.9 | - |
|  | Minimum | 14.4 | - | 0.0 | 0.0 | 0.0 | 827.7 | | - | 0.5 | - |
|  | Completeness | 100.0% | - | 100.0% | 100.0% | 36.4% | 100.0% | | - | 100.0% | - |

| Table S1 (cont’d): Summary of GLDAS- CHIRPS and weather-station-based hydrometeorological variables for the eight MAL-ED study sites, 2009-2014 | | | | | | | | | | | |
| --- | --- | --- | --- | --- | --- | --- | --- | --- | --- | --- | --- |
| Variable | | Relative humidity (%) | | Specific humidity (kg/kg) | | Solar radiation (W/m2) | | Soil moisture (%) | | Surface runoff (mm) | |
|  |  | **GLDAS** | **Station** | **GLDAS** | **Station** | **GLDAS** | **Station** | **GLDAS** | **Station** | **GLDAS** | **Station** |
| Bangladesh | Median | 71.7 | - | 0.016 | - | 202.8 | - | 30.2 | - | 0.0 | - |
|  | IQR | 23.2 | - | 0.010 | - | 64.9 | - | 8.9 | - | 0.0 | - |
|  | Maximum | 97.2 | - | 0.024 | - | 298.4 | - | 39.8 | - | 36.1 | - |
|  | Minimum | 23.1 | - | 0.003 | - | 15.3 | - | 19.1 | - | 0.0 | - |
|  | Completeness | 100.0% | - | 100.0% | - | 100.0% | - | 100.0% | - | 100.0% | - |
| Brazil | Median | 72.7 | - | 0.016 | - | 259.6 | - | 17.0 | - | 0.0 | - |
|  | IQR | 8.0 | - | 0.002 | - | 61.9 | - | 8.5 | - | 0.0 | - |
|  | Maximum | 92.1 | - | 0.019 | - | 338.9 | - | 38.4 | - | 7.7 | - |
|  | Minimum | 58.6 | - | 0.012 | - | 45.8 | - | 13.2 | - | 0.0 | - |
|  | Completeness | 100.0% | - | 100.0% | - | 100.0% | - | 100.0% | - | 100.0% | - |
| India | Median | 64.3 | 69.0 | 0.014 | - | 238.7 | - | 23.8 | - | 0.0 | - |
|  | IQR | 21.8 | 19.5 | 0.003 | - | 70.4 | - | 13.1 | - | 0.0 | - |
|  | Maximum | 95.8 | 99.0 | 0.020 | - | 311.1 | - | 41.6 | - | 31.2 | - |
|  | Minimum | 29.1 | 36.5 | 0.006 | - | 15.0 | - | 12.1 | - | 0.0 | - |
|  | Completeness | 100.0% | 84.6% | 100.0% | - | 100.0% | - | 100.0% | - | 100.0% | - |
| Nepal | Median | 62.6 | - | 0.007 | - | 222.9 | - | 18.7 | - | 0.0 | - |
|  | IQR | 42.5 | - | 0.009 | - | 96.1 | - | 12.9 | - | 0.0 | - |
|  | Maximum | 97.5 | - | 0.017 | - | 350.7 | - | 37.1 | - | 17.4 | - |
|  | Minimum | 4.6 | - | 0.001 | - | 12.8 | - | 9.9 | - | 0.0 | - |
|  | Completeness | 100.0% | - | 100.0% | - | 100.0% | - | 100.0% | - | 100.0% | - |
| Pakistan | Median | 26.9 | 63.5 | 0.006 | - | 236.2 | - | 11.2 | - | 0.0 | - |
|  | IQR | 17.6 | 13.5 | 0.009 | - | 97.4 | - | 4.9 | - | 0.0 | - |
|  | Maximum | 82.8 | 98.0 | 0.021 | - | 322.5 | - | 34.1 | - | 19.8 | - |
|  | Minimum | 4.2 | 27.0 | 0.001 | - | 7.7 | - | 8.1 | - | 0.0 | - |
|  | Completeness | 100.0% | 100.0% | 100.0% | - | 100.0% | - | 100.0% | - | 100.0% | - |
| Peru | Median | 87.3 | - | 0.018 | - | 191.6 | - | 34.1 | - | 0.0 | - |
|  | IQR | 9.8 | - | 0.002 | - | 86.3 | - | 2.1 | - | 0.2 | - |
|  | Maximum | 98.1 | - | 0.021 | - | 313.4 | - | 38.2 | - | 27.5 | - |
|  | Minimum | 54.5 | - | 0.011 | - | 14.8 | - | 28.0 | - | 0.0 | - |
|  | Completeness | 100.0% | - | 100.0% | - | 100.0% | - | 100.0% | - | 100.0% | - |
| South Africa | Median | 73.0 | 67.0 | 0.011 | - | 207.5 | - | 13.5 | - | 0.0 | - |
|  | IQR | 21.5 | 19.0 | 0.006 | - | 91.3 | - | 5.6 | - | 0.0 | - |
|  | Maximum | 97.4 | 97.0 | 0.019 | - | 350.0 | - | 32.6 | - | 4.1 | - |
|  | Minimum | 17.1 | 15.0 | 0.002 | - | 7.4 | - | 8.3 | - | 0.0 | - |
|  | Completeness | 100.0% | 74.4% | 100.0% | - | 100.0% | - | 100.0% | - | 100.0% | - |
| Tanzania | Median | 77.7 | - | 0.012 | - | 274.6 | - | 15.9 | - | 0.0 | - |
|  | IQR | 17.5 | - | 0.003 | - | 65.0 | - | 6.5 | - | 0.0 | - |
|  | Maximum | 97.6 | - | 0.015 | - | 348.5 | - | 32.7 | - | 5.1 | - |
|  | Minimum | 39.0 | - | 0.006 | - | 21.6 | - | 11.5 | - | 0.0 | - |
|  | Completeness | 100.0% | - | 100.0% | - | 100.0% | - | 100.0% | - | 100.0% | - |

| Table S2: Evaluation statistics for hydrometeorological variables from the closest weather stations to each of the eight MAL-ED sites^[[1]](#footnote-1)^ | | | | | | | | | | | | | | | | | | | | | | |
| --- | --- | --- | --- | --- | --- | --- | --- | --- | --- | --- | --- | --- | --- | --- | --- | --- | --- | --- | --- | --- | --- | --- |
| Variable | | BGD | | BRF | | INV | | NEB | | | | PKN | | | PEL | | | SAV | | TZH | | |
|  |  | Daily | 7-day average | Daily | 7-day average | Daily | 7-day average | Daily | | 7-day average | | Daily | | 7-day average | Daily | | 7-day average | Daily | 7-day average | Daily | | 7-day average |
| Median temperature (C) | **n** | 1,307 | | 2,186 | | 1,851 | | 2,138 | | | 2,170 | | | | 2,190 | | | 2,106 | | - | | |
|  | **R** | 0.93 | 0.95 | 0.58 | 0.68 | 0.93 | 0.97 | 0.93 | 0.95 | | 0.99 | | 0.99 | | 0.45 | 0.59 | | 0.87 | 0.96 | - | - | |
|  | **NSE** | 0.85 | 0.89 | 0.05 | 0.24 | 0.76 | 0.84 | 0.55 | 0.58 | | 0.81 | | 0.82 | | -1.22 | -2.28 | | 0.73 | 0.89 | - | - | |
|  | **MBE** | 0.27 | 0.28 | 0.13 | 0.13 | 0.86 | 0.86 | 2.89 | 2.89 | | -2.90 | | -2.90 | | 1.11 | 1.11 | | 0.51 | 0.50 | - | - | |
|  | **RMSE** | 1.54 | 1.27 | 0.88 | 0.65 | 1.58 | 1.25 | 3.51 | 3.32 | | 3.20 | | 3.05 | | 1.82 | 1.43 | | 1.98 | 1.09 | - | - | |
|  | **TPR** | 0.28 | 0.27 | 0.51 | 0.59 | 0.51 | 0.54 | 0.52 | 0.55 | | 0.81 | | 0.83 | | 0.43 | 0.54 | | 0.57 | 0.62 | - | - | |
|  | **FPR** | 0.12 | 0.12 | 0.12 | 0.10 | 0.05 | 0.04 | 0.12 | 0.10 | | 0.05 | | 0.03 | | 0.14 | 0.12 | | 0.09 | 0.07 | - | - | |
| Precipitation (mm) - GLDAS | **n** | 1,306 | | 2,168 | | 1,857 | | 1,830 | | | 2,167 | | | | 1,991 | | | 1,933 | | 798 | | |
|  | **R** | 0.50 | 0.68 | 0.50 | 0.78 | 0.24 | 0.64 | 0.27 | 0.61 | | 0.17 | | 0.57 | | 0.34 | 0.42 | | 0.37 | 0.65 | 0.22 | 0.67 | |
|  | **NSE** | 0.01 | 0.20 | 0.18 | 0.58 | -0.30 | 0.22 | -0.10 | 0.35 | | -0.73 | | -0.62 | | -0.24 | -0.22 | | 0.02 | 0.40 | -0.84 | -0.10 | |
|  | **MBE** | -1.31 | -1.44 | -0.13 | -0.10 | -0.58 | -0.57 | 0.12 | 0.03 | | -0.40 | | -0.42 | | -2.17 | -2.10 | | 0.23 | 0.21 | -0.68 | -0.67 | |
|  | **RMSE** | 12.06 | 6.13 | 7.64 | 2.87 | 9.71 | 3.46 | 9.23 | 4.12 | | 5.63 | | 2.29 | | 14.07 | 5.71 | | 7.74 | 2.96 | 7.03 | 2.44 | |
|  | **TPR** | 0.27 | 0.28 | 0.56 | 0.70 | 0.33 | 0.38 | 0.43 | 0.47 | | 0.47 | | 0.72 | | 0.32 | 0.32 | | 0.37 | 0.45 | 0.19 | 0.20 | |
|  | **FPR** | 0.12 | 0.11 | 0.12 | 0.07 | 0.13 | 0.11 | 0.09 | 0.06 | | 0.11 | | 0.12 | | 0.15 | 0.16 | | 0.11 | 0.10 | 0.22 | 0.21 | |
| Precipitation (mm) - CHIRPS | **n** | 1,306 | | 2,168 | | 1,857 | | 1,830 | | | 2,167 | | | | 1,991 | | | 1,933 | | 798 | | |
|  | **R** | 0.52 | 0.74 | 0.50 | 0.85 | 0.25 | 0.66 | 0.30 | 0.69 | | 0.17 | | 0.47 | | 0.35 | 0.51 | | 0.43 | 0.70 | 0.21 | 0.60 | |
|  | **NSE** | 0.12 | 0.44 | -0.26 | 0.56 | -0.15 | 0.35 | -0.71 | 0.21 | | -0.03 | | 0.20 | | -0.25 | 0.09 | | -0.11 | 0.39 | -1.57 | -1.24 | |
|  | **MBE** | -1.08 | -1.25 | -0.61 | -0.57 | -0.33 | -0.31 | -1.00 | -1.16 | | 0.06 | | 0.06 | | -1.05 | -0.94 | | -0.20 | -0.26 | -1.47 | -1.45 | |
|  | **RMSE** | 11.38 | 5.12 | 9.47 | 2.97 | 9.15 | 3.18 | 11.51 | 4.56 | | 4.35 | | 1.61 | | 14.11 | 4.92 | | 8.21 | 3.00 | 8.31 | 3.48 | |
|  | **TPR** | 0.27 | 0.29 | 0.46 | 0.78 | 0.34 | 0.37 | 0.38 | 0.50 | | 0.38 | | 0.68 | | 0.33 | 0.32 | | 0.27 | 0.44 | 0.13 | 0.17 | |
|  | **FPR** | 0.12 | 0.10 | 0.08 | 0.05 | 0.13 | 0.12 | 0.10 | 0.05 | | 0.06 | | 0.12 | | 0.15 | 0.15 | | 0.08 | 0.10 | 0.18 | 0.26 | |
| Surface pressure (Pa) | **n** | 1,295 | | 1,534 | | - | | 25 | | | | 21 | | | 2,059 | | | 2,080 | | - | | |
|  | **R** | 1.00 | 0.99 | 0.92 | 0.94 | - | - | 0.95 | | 0.87 | | 0.99 | | 0.96 | 0.95 | | 0.97 | 0.88 | 0.83 | - | | - |
|  | **NSE** | 0.96 | 0.96 | -52.71 | -67.65 | - | - | -62.80 | | -70.15 | | 0.96 | | 0.92 | -0.47 | | -1.02 | 0.74 | 0.56 | - | | - |
|  | **MBE** | -0.89 | -0.90 | 12.20 | 12.20 | - | - | 26.77 | | 26.88 | | 1.42 | | 0.64 | 2.53 | | 2.53 | -0.12 | -0.12 | - | | - |
|  | **RMSE** | 1.02 | 1.07 | 12.22 | 12.21 | - | - | 26.79 | | 26.93 | | 1.66 | | 2.47 | 2.62 | | 2.57 | 2.43 | 2.42 | - | | - |
|  | **TPR** | 0.38 | 0.37 | 0.42 | 0.42 | - | - | 0.20 | | 0.20 | | 0.20 | | 0.20 | 0.68 | | 0.71 | 0.66 | 0.64 | - | | - |
|  | **FPR** | 0.01 | 0.01 | 0.03 | 0.03 | - | - | 0.00 | | 0.00 | | 0.00 | | 0.00 | 0.04 | | 0.03 | 0.06 | 0.06 | - | | - |
| Wind speed (m/s) | **n** | 1,300 | | 2,186 | | - | | 2,138 | | | | 2,170 | | | 2,072 | | | 2,106 | | - | | |
|  | **R** | 0.56 | 0.60 | 0.89 | 0.93 | - | - | 0.25 | | 0.32 | | 0.59 | | 0.67 | 0.28 | | 0.24 | 0.55 | 0.45 | - | | - |
|  | **NSE** | -7.19 | -13.63 | 0.78 | 0.84 | - | - | -1.32 | | -1.92 | | -11.29 | | -21.70 | -0.16 | | -0.41 | -0.39 | -1.32 | - | | - |
|  | **MBE** | -1.89 | -1.90 | 0.04 | 0.04 | - | - | -0.46 | | -0.46 | | -2.05 | | -2.05 | 0.24 | | 0.24 | 0.35 | 0.35 | - | | - |
|  | **RMSE** | 2.08 | 2.01 | 0.75 | 0.58 | - | - | 0.92 | | 0.76 | | 2.36 | | 2.27 | 0.58 | | 0.44 | 0.84 | 0.55 | - | | - |
|  | **TPR** | 0.26 | 0.27 | 0.69 | 0.72 | - | - | 0.31 | | 0.36 | | 0.47 | | 0.48 | 0.32 | | 0.33 | 0.49 | 0.38 | - | | - |
|  | **FPR** | 0.10 | 0.13 | 0.06 | 0.06 | - | - | 0.16 | | 0.14 | | 0.13 | | 0.12 | 0.15 | | 0.14 | 0.11 | 0.15 | - | | - |
| Relative humidity (%) | **n** | - | | - | | 1,854 | | - | | | | 2,191 | | | - | | | 1,631 | | - | | |
|  | **R** | - | - | - | - | 0.79 | 0.86 | - | | - | | 0.65 | | 0.72 | - | | - | 0.88 | 0.88 | - | | - |
|  | **NSE** | - | - | - | - | 0.40 | 0.47 | - | | - | | -8.66 | | -10.55 | - | | - | 0.68 | 0.62 | - | | - |
|  | **MBE** | - | - | - | - | 4.62 | 4.61 | - | | - | | 33.19 | | 33.20 | - | | - | -4.16 | -4.19 | - | | - |
|  | **RMSE** | - | - | - | - | 9.67 | 7.92 | - | | - | | 34.59 | | 34.10 | - | | - | 8.60 | 7.00 | - | | - |
|  | **TPR** | - | - | - | - | 0.42 | 0.48 | - | | - | | 0.58 | | 0.65 | - | | - | 0.38 | 0.40 | - | | - |
|  | **FPR** | - | - | - | - | 0.10 | 0.07 | - | | - | | 0.11 | | 0.09 | - | | - | 0.08 | 0.06 | - | | - |

| Table S3: Evaluation statistics for key hydrometeorological variables during peak season from the closest weather stations to each of the eight MAL-ED sites^[[2]](#footnote-2)^ | | | | | | | | | | | | | | | | | | | |
| --- | --- | --- | --- | --- | --- | --- | --- | --- | --- | --- | --- | --- | --- | --- | --- | --- | --- | --- | --- |
| Variable | | BGD | | BRF | | INV | | NEB | | PKN | | | PEL | | SAV | | | TZH | |
|  |  | Daily | 7-day average | Daily | 7-day average | Daily | 7-day average | Daily | 7-day average | Daily | 7-day average | Daily | | 7-day average | Daily | 7-day average | | Daily | 7-day average |
| Peak season | | Nov - Feb | | Sep - Nov | | Dec - Mar | | Dec - Feb | | Nov - Feb | | | Mar - Jun | | Feb - Jun | | | May - Jul | |
| Median temperature (C) | **n** | 364 | | 545 | | 622 | | 1,756 | | 704 | | | 732 | | 877 | | | - | |
|  | **R** | 0.89 | 0.93 | 0.40 | 0.56 | 0.90 | 0.95 | 0.95 | 0.97 | 0.95 | 0.98 | | 0.42 | 0.64 | 0.87 | | 0.96 | - | - |
|  | **NSE** | 0.77 | 0.82 | -0.41 | -0.22 | 0.56 | 0.58 | 0.55 | 0.58 | 0.47 | 0.54 | | -2.20 | -5.83 | 0.69 | | 0.83 | - | - |
|  | **MBE** | 0.18 | 0.28 | -0.15 | -0.15 | 1.21 | 1.20 | 3.30 | 3.30 | -2.44 | -2.42 | | 1.48 | 1.48 | 0.89 | | 0.89 | - | - |
|  | **RMSE** | 1.41 | 1.12 | 0.71 | 0.50 | 1.63 | 1.37 | 3.77 | 3.60 | 2.73 | 2.52 | | 1.97 | 1.62 | 1.93 | | 1.25 | - | - |
|  | **TPR** | 0.27 | 0.28 | 0.35 | 0.50 | 0.59 | 0.63 | 0.53 | 0.56 | 0.86 | 0.88 | | 0.39 | 0.56 | 0.60 | | 0.71 | - | - |
|  | **FPR** | 0.09 | 0.07 | 0.14 | 0.12 | 0.03 | 0.01 | 0.10 | 0.09 | 0.04 | 0.02 | | 0.15 | 0.11 | 0.09 | | 0.05 | - | - |
| Precipitation (mm) - GLDAS | **n** | 364 | | 544 | | 626 | | 1,487 | | 704 | | | 665 | | 817 | | | 111 | |
|  | **R** | 0.50 | 0.52 | 0.10 | 0.16 | 0.15 | 0.65 | 0.27 | 0.60 | -0.01 | 0.34 | | 0.30 | 0.34 | 0.34 | | 0.66 | -0.04 | -0.06 |
|  | **NSE** | 0.24 | 0.23 | -1.12 | -1.29 | -0.21 | 0.37 | -0.10 | 0.35 | -2.58 | -2.67 | | -0.37 | -0.40 | -0.12 | | 0.41 | -18.17 | -9.88 |
|  | **MBE** | 0.06 | 0.06 | -0.01 | -0.04 | 0.21 | 0.16 | 0.24 | 0.16 | -0.06 | -0.06 | | -3.13 | -2.92 | 0.04 | | 0.05 | -0.42 | -0.43 |
|  | **RMSE** | 2.47 | 1.55 | 1.20 | 0.57 | 5.91 | 1.99 | 10.04 | 4.49 | 0.99 | 0.36 | | 16.27 | 6.61 | 5.66 | | 2.22 | 3.43 | 1.48 |
|  | **TPR** | 0.14 | 0.27 | 0.24 | 0.41 | 0.31 | 0.39 | 0.41 | 0.44 | 0.14 | 0.63 | | 0.30 | 0.31 | 0.39 | | 0.45 | 0.14 | 0.18 |
|  | **FPR** | 0.11 | 0.10 | 0.12 | 0.14 | 0.15 | 0.12 | 0.09 | 0.07 | 0.06 | 0.16 | | 0.16 | 0.16 | 0.12 | | 0.10 | 0.16 | 0.27 |
| Precipitation (mm) - CHIRPS | **n** | 364 | | 544 | | 626 | | 1,487 | | 704 | | | 665 | | 817 | | | 111 | |
|  | **R** | 0.21 | 0.72 | 0.06 | 0.09 | 0.06 | 0.51 | 0.29 | 0.68 | -0.01 | 0.31 | | 0.29 | 0.45 | 0.31 | | 0.62 | 0.05 | -0.02 |
|  | **NSE** | -0.01 | 0.34 | -0.26 | -0.24 | -0.39 | 0.19 | -0.74 | 0.19 | -0.27 | 0.01 | | -0.35 | 0.04 | -0.52 | | 0.28 | -8.49 | -4.24 |
|  | **MBE** | 0.06 | 0.08 | 0.15 | 0.16 | 0.36 | 0.34 | -1.12 | -1.31 | 0.01 | 0.01 | | -1.29 | -1.05 | -0.13 | | -0.14 | -0.30 | -0.29 |
|  | **RMSE** | 2.85 | 1.43 | 0.92 | 0.42 | 6.35 | 2.26 | 12.59 | 5.00 | 0.59 | 0.19 | | 16.16 | 5.49 | 6.58 | | 2.44 | 2.41 | 1.03 |
|  | **TPR** | 0.06 | 0.23 | 0.04 | 0.25 | 0.10 | 0.38 | 0.38 | 0.47 | 0.07 | 0.54 | | 0.31 | 0.28 | 0.23 | | 0.42 | 0.02 | 0.13 |
|  | **FPR** | 0.04 | 0.16 | 0.01 | 0.04 | 0.04 | 0.11 | 0.10 | 0.06 | 0.02 | 0.07 | | 0.15 | 0.17 | 0.05 | | 0.11 | 0.04 | 0.29 |
| Peak season | | Nov - Feb | | Sep - Nov | | Dec - Mar | | Dec - Feb | | Nov - Feb | | | Mar - Jun | | Feb - Jun | | | May - Jul | |
| Surface pressure (Pa) | **n** | 362 | | 405 | | - | | 24 | | 4 | | | 669 | | 869 | | | - | |
|  | **R** | 0.98 | 0.95 | 0.95 | 0.96 | - | - | 0.95 | 0.85 | 0.97 | 0.93 | 0.92 | | 0.95 | 0.86 | 0.78 | | - | - |
|  | **NSE** | 0.82 | 0.67 | -56.01 | -69.72 | - | - | -65.65 | -74.68 | 0.77 | 0.80 | -1.12 | | -2.48 | 0.66 | 0.39 | | - | - |
|  | **MBE** | -0.90 | -0.99 | 12.47 | 12.44 | - | - | 26.74 | 26.85 | 1.29 | -0.61 | 2.35 | | 2.35 | 0.22 | 0.22 | | - | - |
|  | **RMSE** | 1.02 | 1.19 | 12.48 | 12.45 | - | - | 26.76 | 26.90 | 1.53 | 1.40 | 2.45 | | 2.39 | 2.53 | 2.54 | | - | - |
|  | **TPR** | 0.32 | 0.30 | 0.45 | 0.43 | - | - | 0.20 | 0.20 | 0.20 | 0.20 | 0.59 | | 0.61 | 0.63 | 0.62 | | - | - |
|  | **FPR** | 0.03 | 0.05 | 0.03 | 0.04 | - | - | 0.00 | 0.00 | 0.25 | 0.25 | 0.06 | | 0.05 | 0.08 | 0.08 | | - | - |
| Wind speed (m/s) | **n** | 363 | | 545 | | - | | 1,756 | | 704 | | | 715 | | 877 | | | - | |
|  | **R** | 0.50 | 0.64 | 0.80 | 0.81 | - | - | 0.14 | 0.16 | 0.58 | 0.64 | 0.35 | | 0.40 | 0.53 | 0.41 | | - | - |
|  | **NSE** | -13.41 | -31.06 | 0.53 | 0.50 | - | - | -1.53 | -2.26 | -10.34 | -25.42 | -0.35 | | -1.03 | -0.46 | -1.73 | | - | - |
|  | **MBE** | -1.68 | -1.70 | 0.37 | 0.36 | - | - | -0.43 | -0.42 | -1.38 | -1.37 | 0.34 | | 0.34 | 0.46 | 0.44 | | - | - |
|  | **RMSE** | 1.79 | 1.74 | 0.84 | 0.72 | - | - | 0.89 | 0.72 | 1.51 | 1.41 | 0.58 | | 0.45 | 0.83 | 0.59 | | - | - |
|  | **TPR** | 0.25 | 0.28 | 0.61 | 0.79 | - | - | 0.25 | 0.32 | 0.58 | 0.66 | 0.39 | | 0.33 | 0.47 | 0.45 | | - | - |
|  | **FPR** | 0.08 | 0.08 | 0.09 | 0.05 | - | - | 0.16 | 0.17 | 0.09 | 0.08 | 0.14 | | 0.15 | 0.10 | 0.13 | | - | - |
| Relative humidity (%) | **n** | - | | - | | 625 | | - | | 709 | | | - | | 625 | | | - | |
|  | **R** | - | - | - | - | 0.79 | 0.83 | - | - | 0.54 | 0.65 | - | | - | 0.83 | 0.80 | | - | - |
|  | **NSE** | - | - | - | - | 0.49 | 0.55 | - | - | -27.13 | -48.15 | - | | - | 0.44 | 0.23 | | - | - |
|  | **MBE** | - | - | - | - | 2.38 | 2.42 | - | - | 38.74 | 38.81 | - | | - | -6.13 | -6.28 | | - | - |
|  | **RMSE** | - | - | - | - | 7.60 | 6.16 | - | - | 39.69 | 39.22 | - | | - | 9.76 | 8.69 | | - | - |
|  | **TPR** | - | - | - | - | 0.41 | 0.44 | - | - | 0.44 | 0.55 | - | | - | 0.35 | 0.40 | | - | - |
|  | **FPR** | - | - | - | - | 0.11 | 0.10 | - | - | 0.14 | 0.11 | - | | - | 0.08 | 0.04 | | - | - |

1. n = number of observations; R = correlation coefficient; NSE = Nash-Sutcliffe efficiency coefficient; MBE = mean bias error; RMSE = Root mean square error; TPR/FPR = True/false positive rate (for days exceeding the 80^th^ percentile); BGD = Dhaka, Bangladesh; BRF = Fortaleza, Brazil; INV = Vellore, India; PKN = Naushero Feroze, Pakistan; PEL = Loreto, Peru; SAV = Venda, South Africa; TZH = Haydom, Tanzania. [↑](#footnote-ref-1)
2. n = number of observations; R = correlation coefficient; NSE = Nash-Sutcliffe efficiency coefficient; MBE = mean bias error; RMSE = Root mean square error; TPR/FPR = True/false positive rate (for days exceeding the 80^th^ percentile); BGD = Dhaka, Bangladesh; BRF = Fortaleza, Brazil; INV = Vellore, India; PKN = Naushero Feroze, Pakistan; PEL = Loreto, Peru; SAV = Venda, South Africa; TZH = Haydom, Tanzania. [↑](#footnote-ref-2)
